# Stochastic analysis of compact stars under composite polytropes

**DOI:** 10.1038/s41598-026-53379-6

**Published:** 2026-06-09

**Authors:** Mohamed I. Nouh, Samah H. El-Essawy, Mona M. Foda, Mohamed S. Aboueisha

**Affiliations:** 1 Astronomy Department, National Research Institute of Astronomy and Geophysics, Helwan, Cairo, 11421 Egypt; 2 Astronomy Department, Faculty of Science, Cairo University, Giza, Egypt 12613

**Keywords:** Compact stars, Mass-radius relation, Monte Carlo methods, Parameter estimation, Astronomy and planetary science, Mathematics and computing, Physics

## Abstract

The study of dense matter has been greatly advanced by progress in theoretical high-energy simulations and modern observational astronomy. Neutron stars serve as natural laboratories for probing matter under extreme density and strong gravitational fields. While polytropic equations of state are widely employed, conventional models based on a single polytropic index cannot adequately represent the stratified, layered nature of realistic compact-star interiors. To address this limitation, we develop a composite relativistic polytropic model in which the polytropic index varies smoothly with radius. By coupling the Einstein field equations with a generalized composite polytropic equation of state, we derive the composite Tolman–Oppenheimer–Volkoff (CTOV) system. The resulting nonlinear equations are solved using a Monte Carlo-based numerical integration method, which efficiently handles stiffness while enabling probabilistic exploration of the parameter space and natural uncertainty quantification. Our results demonstrate that increasing the relativistic parameter σ significantly reduces both the Emden function and the enclosed mass function, producing more compact stellar configurations. Sharper core–envelope transitions (ε = 0.01) yield systematically higher compactness than smoother transitions (ε = 0.03). The derived mass–radius relations reproduce the observed diversity of neutron stars, successfully matching both low-mass, large-radius systems such as PSR J0030 + 0451 and high-mass compact pulsars such as PSR J1614–2230. Importantly, the maximum-mass analysis shows that stiff composite configurations (n_c_ = 1, n_e_ = 2, x_c_ = 0.7) can support gravitational masses up to M_max_ ≈ 3.47 M$_{ \odot }$ for ε = 0.01 and M_max_ ≈ 2.71 M$_{ \odot }$ for ε = 0.03, with corresponding minimum radii in the range R_min_ ≈ 10.6–13.2 km, consistent with current observational constraints. These findings confirm that composite polytropes provide a flexible, physically motivated framework for modeling stratified compact stars and for constraining the dense-matter equation of state.

## Introduction

Extreme physics (from the strongest gravitational attraction to the outflow of matter at speeds near the speed of light) and cutting-edge observations converge in the astrophysics of compact objects, such as black holes, neutron stars, and white dwarfs. These dense remnants provide natural laboratories for testing high-energy processes, nuclear physics, and gravity theories by pushing matter, energy, and spacetime to their limits^1–4^. An essential ingredient in the development of these models is the equation of state (EoS), which relates pressure to density in the stellar interior. The polytropic relation $\:P=K{{\uprho\:}}^{1+1/n}$ is a fundamental parametric EoS in stellar astrophysics, valued for its simplicity and physical clarity^5–8^. In this equation, $\:n$ represents the polytropic index, while $\:K$ is a constant dependent on composition and entropy. Polytropic models have been widely utilised in both Newtonian and broad relativistic contexts to investigate the equilibrium structure, stability, and scaling relations of stars^9–15^.

Although a single polytropic index can yield valuable insights into the overarching characteristics of idealised stellar models, real compact stars exhibit significant internal stratification. A neutron star is not a uniform sphere; its interior consists of stratified layers, each regulated by varying microphysical processes. This limitation mandates the use of composite or multi-polytropic models, in which the polytropic index is permitted to vary with the radial coordinate $\:r$, effectively stitching together distinct EoS segments to represent the layered interior^16–20^.

For a star approximated with a composite polytropic EoS, these become the CTOV equations, which are a set of coupled, nonlinear ordinary differential equations^21^. Solving the CTOV system analytically is often intractable, except in rare circumstances, making numerical integration crucial. However, traditional numerical solvers (e.g., Runge-Kutta methods) confront numerous obstacles in this setting. The equations are often stiff, especially at the center and surface, and solutions can be very sensitive to the parameter choices, such as the central density $\:{{\uprho\:}}_{c}$, the sharpness of internal transitions, and the relativistic parameter$\:\:{\upsigma\:}$. Furthermore, classic deterministic approaches provide a single solution trajectory but offer no inherent framework for quantifying uncertainty in model predictions, which is critical when comparing model predictions to observations that have their own measurement errors. This is particularly important given the well-known degeneracies in the mass-radius relation, where very different internal equations of state can yield similar global observables.

Additional physical complexity derives from the possibility of pressure anisotropy within compact stars. In a perfect fluid, pressure is isotropic, but in actual settings, high magnetic fields, phase transitions, solid crusts, superfluidity, or pion/kaon condensates can lead to large discrepancies between the radial pressure $\:{(P}_{r})$ and the tangential pressure $\:\left({P}_{t}\right)$^9–12,22–26^. This anisotropy alters the hydrostatic equilibrium condition, leading to an anisotropic version of the TOV equation.

While we focus here on the isotropic scenario, the framework we propose might be extended to include such effects, which are relevant for simulating magnetars, solid planetary cores, or stars with superfluid vortices^27,28^. To address the challenges of solving the CTOV system while robustly exploring parameter space and quantifying uncertainties, we turn to stochastic numerical methods.

MC algorithms are designed to sample from complex, high-dimensional probability distributions, making them excellent for issues where parameters are degenerate, and likelihood surfaces are intricate^29–32^. In astrophysics, MC has become a standard tool for parameter estimation and model comparison in disciplines ranging from cosmology and exoplanet discovery to gravitational-wave astronomy. Implementations like the affine-invariant ensemble sampler (emcee) have substantially enhanced the efficiency and accessibility of MC for the community^30,35^. Recent methodological improvements continue to expand its utility, including methods for estimating marginal likelihoods directly from MC outputs^33^ and studies of quicker alternatives, such as variational inference, for specific situations^34–38^.

The application of MC methods to the direct numerical solution of differential equations in astrophysics has been pioneered in recent work. El-Essawy et al.^39,40^ devised two distinct MC algorithms as a versatile numerical solver for the Lane-Emden equations governing Newtonian polytropes. Nouh et al.^41^ and El-Essawy et al.^42^ extended the MC calculations to the polytropic and isothermal gas spheres and to Lane-Emden equations with cylindrical and planar symmetries, confirming the method’s scalability and resilience across a range of polytropic indices and geometries.

In the present study, we build on this basis by utilising an MC-based technique to solve the relativistic CTOV equations for composite polytropes. Our model incorporates a smoothly evolving polytropic index $\:n\left(r\right)$, parameterised to depict a star with a separate core and envelope. The MC algorithm is employed not just for posterior parameter estimation after solving the equations, but as the core integration engine itself. By treating the integration of the differential equations as a stochastic sampling process, we obtain robust numerical solutions for the density, pressure, and mass profiles and probabilistically explore the parameter space. This allows us to systematically investigate the influence of key physical parameters, central density $\:\left({{\uprho\:}}_{c}\right)$, core boundary $\:\left(\:{r}_{c}\:\right)$, transition width $\:\left(\:\right)$, and the relativistic parameter $\:\left(\:{\upsigma\:}\right)$, on the resulting stellar structure and global properties like total mass $\:\left(\:M\:\right)$ and radius $\:\left(\:R\:\right)$.

The primary goals of this study are threefold: (1) to demonstrate the efficacy of the MC method as a solver for the complex, nonlinear CTOV system; (2) to quantify how the choice of composite model parameters affects the predicted mass-radius relation and internal structure of stratified compact objects; and (3) to provide a framework that naturally outputs uncertainties on these predictions, reflecting the sensitivity of the models to input assumptions. In particular, this approach allows for us to derive, for the first time in this CTOV context, systematic trends and internal error budgets for key structural diagnostics such as the first zero of the Emden function, the mass function, and the compactness, as well as probabilistic constraints on core size and mass that go beyond the single best-fit values reported in earlier deterministic studies.

The paper is organised as follows. Section  2 introduces the composite CTOV formulation with a smoothly varying polytropic index. Section  3 presents the numerical methodology based on the Monte Carlo integration scheme. Section  4 provides a detailed physical analysis of the composite polytropic models, including the behavior of the Emden and mass functions, and the effects of the relativistic parameter and transition width on stellar structure. Section  5 discusses the stability analysis of the models, covering the adiabatic index, the causality condition, the Zeldovich condition, and the energy conditions. Section  6 presents the mass–radius relation, compares theoretical predictions with observational data for 21 neutron stars. Section  7 presents the maximum-mass analysis for both the soft and stiff composite models and comparison with key observational benchmarks. Finally, Sect.  8 summarises the study’s main conclusions.

## Field equations and formalism

In this section, we present the general relativistic framework governing the structure of compact stars and derive the Tolman–Oppenheimer–Volkoff (TOV) equations. We then extend this formalism to incorporate a composite polytropic equation of state with a smoothly varying radial polytropic index, leading to the CTOV system used throughout this work. A detailed derivation of the composite relativistic polytropic equations, originally presented by^21^.

### Einstein field equations and TOV system

We consider a static, spherically symmetric spacetime described by the line element:

1 $$\:d{s}^{2}={e}^{2\:{\uppsi\:}\left(r\right)}{c}^{2}d{t}^{2}-{e}^{2{\Lambda\:}\left(r\right)}d{r}^{2}-{r}^{2}d{{\Omega\:}}^{2},$$

where ψ(r) and Λ(r) are metric functions depending only on the radial coordinate r.

Assuming the matter inside the star behaves as an isotropic perfect fluid, the energy–momentum tensor is given by

2 $$\:{T}^{\alpha\:\beta\:}\:=\:\left(\rho\:\:+\frac{P}{{c}^{2}}\right){u}^{\alpha\:}\:{u}^{\beta\:}\:-\:P\:{g}^{\alpha\:\beta\:},$$

where ρ is the energy density, P is the pressure, and $\:{u}^{\alpha\:}$ is the four-velocity of the fluid satisfying $\:{u}^{\alpha\:}\:{u}_{\alpha\:\:}=\:1$.

The Einstein field equations for the energy-momentum tensor (2) and the metric (1) can be expressed as

3 $${e^{ - 2\Lambda }}\left( {\frac{{2\Lambda '}}{r} - \frac{1}{{{r^2}}}} \right)+\frac{1}{{{r^2}}}=\frac{{8\pi G}}{{{c^2}}}\rho$$

4 $${e^{ - 2\Lambda }}(\frac{1}{{{r^2}}}+\frac{{2\psi '}}{r}) - \frac{1}{{{r^2}}}=\frac{{8\pi G}}{{{c^4}}}P$$

5 $${e^{ - 2\Lambda }}\left( {\psi ''+\psi {'^2} - \psi '\Lambda '+\frac{1}{r}\left( {\psi ' - \Lambda '} \right)} \right)=\frac{{8\pi G}}{{{c^4}}}P$$

where $\:G$ is the gravitational constant and $\:c$ is the speed of light and (′) denotes derivation with respect to r. From the conservation of the energy–momentum tensor $T^{{\alpha \beta }} ;\beta = 0$ one obtains

6 $$\left( {\rho {c^2}+P} \right)\frac{{d\psi }}{{dr}}= - \frac{{dP}}{{dr}}.$$

From (3) we get

7 $${e^{2\Lambda }}={\left( {1 - \frac{{2G\,m(r)}}{{{c^2}r}}} \right)^{ - 1}},$$

where $\:m\left(r\right)$ is the gravitational mass enclosed in a sphere with radius $\:r\:$and is given by

8 $$m(r)=\int\limits_{0}^{r} {4\pi \,{{r^{\prime}}^2}\rho dr}$$

From (4), we can write

9 $$\frac{{d\Psi }}{{dr}}=\frac{{\frac{G}{{{c^2}}}m(r)+\frac{{4\pi G}}{{{c^4}}}P{r^3}}}{{r\left( {r - \frac{{2G}}{{{c^2}}}m(r)} \right)}}$$

By inserting Eq. (9) in (6), the TOV equation can be written as

10 $$\:\frac{dP\:}{dr}=\:-\:\frac{\left(\rho\:{c}^{2}+\:P\right)\left(\:\frac{Gm\left(r\right)}{{c}^{2}}+\:\frac{4\pi\:{r}^{3}GP}{{c}^{4}}\right)}{r\:\left(r\:-\:\frac{2Gm\left(r\right)}{{c}^{2}}\:\right)}\:,$$

which expresses the condition of hydrostatic equilibrium in general relativity. The enclosed gravitational mass m(r) is defined by

11 $$\:\frac{dm}{dr}\:=\:4\pi\:{r}^{2}\rho\:.$$

Together, these equations form the standard relativistic description of compact stars.

### Derivation of the CTOV system

To model the internal structure of compact stars, we adopt a generalised polytropic equation of state of the form^6^

12 $$\:P=K{\rho\:}^{\gamma\:\left(r\right)},$$

where ($\:K$) is a constant and $\:\gamma\:\left(r\right)=1+1/n\left(r\right)$ is the adiabatic index, expressed in terms of a radially dependent polytropic index $\:n\left(r\right)$. The central density ($\:{\rho\:}_{c}$) normalizes the density ($\:\rho\:\left(r\right)$) through the Emden function $\:\left(r\right):$

13 $$\rho \:\left( r \right) = \rho \:_{c} \left( r \right)^{{n\left( r \right)}} .$$

The function $\:\left(r\right)$ satisfies $\:\left(0\right)=1$ and decreases outward, vanishing at the stellar surface. Using (13), the polytropic EoS will be in the following form

14 $$P=k{\rho _c}^{{1+\frac{1}{n}}}{\theta ^{n+1}}$$

To model stars with layered structures, we adopt a smoothly varying polytropic index of the form^43^:

15 $$n\left( r \right) = \eta \:_{0} - \eta _{1} \,\tanh \left( {\frac{{r - r_{c} }}{{\varepsilon \:}}} \right),$$

where $\:({{\upeta\:}}_{0}=\left({n}_{c}+{n}_{e}\right)/2)$, $\:({{\upeta\:}}_{1}={n}_{c}-{{\upeta\:}}_{0})$, $\:\left({n}_{c}\right)\:\mathrm{a}\mathrm{n}\mathrm{d}\:\left({n}_{e}\right)$ are the core and envelope polytropic indices, $\:{r}_{c}$ is the core boundary, and $\varepsilon$ is the transition width. This functional form ensures a continuous and differentiable change in the equation of state throughout the stellar interior.

Differentiating the EoS (14) with respect to $\:r$ while keeping $\:n$ variable gives

16 $$\frac{{dP}}{{dr}}=k{\text{ }}{\theta ^{n+1}}{\text{ }}\rho _{c}^{{1+\frac{1}{n}}}{\text{ }}\left( {\left( {\ln \left( \theta \right)\, - {\text{ }}\frac{{\ln \left( {{\rho _c}} \right)}}{{{n^2}}}} \right){\text{ }}\frac{{dn}}{{dr}}{\text{ + }}\frac{{n+1}}{\theta }{\text{ }}\frac{{d\theta }}{{dr}}} \right)\,$$

where

17 $$\frac{{dn}}{{dr}} = \frac{{\eta \:_{1} }}{{\varepsilon \:}}\mathrm{sech} ^{2} \left( {\frac{{r - r_{c} }}{\varepsilon }} \right).$$

18 $$\frac{{d\rho _{c}^{{1+\frac{1}{n}}}}}{{dr}}={\text{ }} - \frac{{\rho _{c}^{{1+\frac{1}{n}}}\ln \left( {{\rho _c}} \right)\,}}{{{n^2}}}{\text{ }}\frac{{dn}}{{dr}}$$

and

$$\frac{{d{\theta ^{n+1}}}}{{dr}}={\theta ^{n+1}}\left( {\ln \left( \theta \right){\text{ }}\frac{{dn}}{{dr}}+\frac{{n+1}}{\theta }{\text{ }}\frac{{d\theta }}{{dr}}} \right).$$

Inserting (13), (14), and (16) into (6) yields

19 $$\:\left({\rho\:}_{c\:}{\theta\:}^{n}{c}^{2}+k\:{\rho\:}_{c}^{1+\frac{1}{n}}\:{\theta\:}^{n+1}\right)\:\frac{d\psi\:\:}{dr}=\:-\:\:k\:{\rho\:}_{c}^{1+\frac{1}{n}}\:{\theta\:}^{n+1}\:\left(\left(\mathrm{ln}\left(\theta\:\right)-\:\frac{\mathrm{ln}\left({\rho\:}_{c}\right)}{{n}^{2}}\right)\:\frac{dn}{dr}+\:\frac{n+1}{\theta\:}\:\frac{d\theta\:}{dr}\right),$$

then, we can write

20 $$\frac{{d\psi }}{{dr}}= - \frac{{\sigma {\text{ }}\theta }}{{\left( {1+\sigma {\text{ }}\theta } \right)}}\,\left( {\left( {\ln \left( \theta \right) - \frac{{\ln \left( {{\rho _c}} \right)}}{{{n^2}}}} \right){\text{ }}\frac{{dn}}{{dr}}+\frac{{\left( {n+1} \right){\text{ }}}}{\theta }\frac{{d\theta }}{{dr}}} \right)$$

where $\sigma$ is the relativistic parameter and is given by

21 $$\sigma =\frac{{{P_c}}}{{{\rho _c}{c^2}}}=\frac{{k\rho _{c}^{{\frac{1}{n}}}}}{{{c^2}}}.$$

Using (7), (14) and (20), Eq. (4) can be written as follows

22 $$\begin{gathered} \frac{{\sigma \left( {n+1} \right)r}}{{\left( {1+\sigma {\text{ }}\theta } \right)}}\left( {1 - \frac{{2G\,m(r)}}{{{c^2}r}}} \right)\frac{{d\theta }}{{dr}}+\frac{{G\,m(r)}}{{{c^2}r}}+\frac{{\sigma {\text{ }}\theta {\text{ }}r}}{{\left( {1+\sigma {\text{ }}\theta } \right)}}\,\left( {\ln \left( \theta \right) - \frac{{\ln \left( {{\rho _c}} \right)}}{{{n^2}}}} \right)\left( {1 - \frac{{2G\,m(r)}}{{{c^2}r}}} \right)\,\frac{{dn}}{{dr}} \hfill \\ \,\,\,\,\,\,\,\,+\,\frac{G}{{{c^2}}}\sigma {\text{ }}\theta \frac{{dm}}{{dr}}=0\,\,\,\, \hfill \\ \end{gathered}$$

where

23 $$\frac{{dm\left( r \right)}}{{dr}}=4\pi {r^2}{\rho _c}{\theta ^n}.$$

To facilitate numerical integration, we introduce dimensionless variables:

24 $$\:\begin{array}{c}x=\frac{r}{L},\:\:\left(x\right)=\frac{\mathrm{m}\left(r\right)}{{M}_{0}},\:\end{array}$$

where the characteristic length $\:L$ and mass scale $\:{M}_{0}$ are given by

25 $$L = \left[ {\frac{{\left( {n_{c} + 1} \right)K\rho _{c}^{{1/n_{c} }} }}{{4\pi G}}} \right]^{{1/2}} ,$$

26 $$M_{0} = 4\pi \:\rho \:_{c} L^{3} .$$

In dimensionless form, the CTOV system becomes

27 $$\:\frac{{x}^{2}}{\left(1+\:\:\sigma \theta\:\right)}\left(1-\:\frac{2\:\:\sigma \left(n+1\right) \nu}{x}\right)\frac{d\theta\:\:}{dx}+\:\frac{\theta\:\:{x}^{2}}{\left(1+ \sigma \theta\:\right)\left(n+1\right)}\:\left(\mathrm{ln}\theta\:-\:\frac{\mathrm{ln}{\rho}_{c}}{{n}^{2}}\right)\left(1-\:\frac{2\:\:\sigma \left(n+1\right)\nu}{x}\:\right)\frac{dn\:}{dx}+\: \nu+\:\sigma\theta\:\:x\:\frac{d\: \nu}{dx}=0,$$

28 $$\frac{{dv}}{{dx}} = x^{2} \theta ^{n} ,$$

with boundary conditions $\theta\:\left(0\right)=1$, $\nu \:\left(0\right)=0$, and $\theta \:\left({x}_{s}\right)=0$ at the surface $\:{x}_{s}$.

The dimensionless system Eqs. (27),(28) depend on the physical parameter set

29 $$\:\Theta = \left\{ {\rho _{c} ,n_{c} ,n_{e} ,x_{c} ,,\sigma } \right\}\:.$$

The model parameters are treated as stochastic variables sampled from predefined, physically motivated ranges, rather than as quantities inferred from a Bayesian posterior. These ranges are selected based on astrophysical constraints (e.g., $\:{n}_{c},{n}_{e}\in\:\left[\mathrm{0.5,2}\right]$, $\varepsilon \in \:\left[ {{\mathrm{0}}{\mathrm{.01,0}}{\mathrm{.03}}} \right]$, $\:\sigma\:\in\:\left[\mathrm{0,0.5}\right]$). The Monte Carlo algorithm uniformly samples the radial coordinate $\:x\:$from a prescribed physically admissible range. For each sampled value, Eqs. (27) and (28) are numerically integrated to obtain the corresponding profiles of $\:\theta\:\:$and $\:\nu\:$. The resulting configurations are then used to construct the mass–radius relation, which is subsequently evaluated against observational constraints or stability criteria to identify physically viable solutions.

This parameterisation allows the stochastic exploration of structurally distinct yet physically plausible stellar configurations, directly incorporating uncertainties in the internal composition of compact objects.

## Numerical methodology – Monte Carlo integration of the CTOV equations

The CTOV system is stiff, nonlinear, and sensitive to input parameters. Traditional deterministic solvers (e.g., Runge–Kutta) provide a single trajectory without uncertainty quantification. We therefore employ a Monte Carlo (MC) integration method that treats the integration as a stochastic sampling process, yielding ensemble solutions and natural error estimates. The general theory of MC integration is provided in Appendix A.

### Advantages of the Monte Carlo method for CTOV

The MC method offers three specific advantages over deterministic integrators for this problem:

1. **Uncertainty quantification**: MC produces an ensemble of solutions from multiple independent runs, providing mean profiles and standard deviations. Runge–Kutta gives only a single solution with no inherent error estimate for parameter sensitivity.
2. **Robustness to stiffness**: The CTOV equations become increasingly stiff near the stellar surface (θ$\:\:\to\:0$) and for sharp transitions ($\varepsilon$= 0.01). The stochastic sampling of the hit-or-miss method avoids the oscillatory instabilities that can plague Runge–Kutta in these regimes.
3. **Natural parameter exploration**: By treating physical parameters as random variables with priors, MC enables probabilistic exploration of degenerate parameter spaces. This is particularly valuable for the mass–radius relation, where different EoS can produce similar observables.

### MC integration scheme

We solve the initial value problem defined by Eqs. (27),(28) using a hit-or-miss MC method adapted for ODEs. The dimensionless radius $\:x$ is discretised with a step $\:\varDelta\:\:x\:=\:0.01$. At each step $\:k$, given $\:{\theta\:}_{k}$ and $\:{\nu}_{k}$, we:

1. Compute the derivatives $\:d\theta\:/dx$ and $\:d\nu/dx$ from the CTOV equations at ($\:{x}_{k}$, $\:{\theta\:}_{k}$, $\:{\nu}_{k}$).
2. For each derivative, pre-generate $\:N\:=\:{2\times\:10}^{6}$ uniform random samples within predetermined bounds $\:[R,\:M]$, where $\:R$ and $\:M$ are the estimated lower and upper bounds of the derivative over the integration domain.
3. Count the number $\:S$ of samples that lie below the derivative (if $\:\frac{d\theta\:}{dx}\ge\:0$) or above the derivative (if $\:\frac{d\theta\:}{dx}\le\:0$).
4. Update the variable using the hit-or-miss rule:

- If $$\:\frac{{d\theta \:}}{{dx}} \ge \:\:0\::\:\theta \:_{{K + 1}} \: = \:\theta \:_{K} \: + \:M\: \cdot \:(S/N) \cdot \Delta x.$$
- If $$\:\frac{{d\theta \:}}{{dx}} \le \:\:0\::\:\theta \:_{{K + 1}} \: = \:\theta \:_{K} \: + \:R\: \cdot \:(S/N) \cdot \Delta x.$$
- The same procedure is applied to $\:\:\nu \left( x \right).$

5. Update the local polytropic index $\:n\left(x\right)$ using the hyperbolic tangent profile (Eq. 15).
6. Terminate the integration when $\:\theta$ becomes negative which defines the stellar surface $\:{x}_{s}.$.

The bounds of $\:M$ and $\:R$ are estimated adaptively from the maximum and minimum expected derivative values over the integration domain. Random samples are generated once per simulation to reduce computational cost.

### Ensemble generation and parameter sampling

We treat the physical parameters $\:({\rho}_{c},{\:n}_{c},{\:n}_{e},\:{x}_{c},\varepsilon\:,\:\sigma\:)$ as random variables with physically motivated priors: $\:{n}_{c},\:{n}_{e}\in\:\:[0.5,\:2]$, $\:\varepsilon\:\in\:[0.01,\:0.03]$, $\sigma\:\:\in\:[0,\:0.5]$, and $\:{\rho}_{c}\:=\:{10}^{15}\:g\:{cm}^{-3}$ fixed for this study. For each parameter combination, we perform $\:m\:=\:10$ independent MC runs, each with 100 candidate surface positions $\:{x}_{s}$ sampled uniformly. The best-fit trajectory for each run is the one in which the final value of θ is closest to zero.

All trajectories are interpolated onto a common 1000-point radial grid $\:x\left( {grid} \right)$ using linear interpolation. For each grid point, we compute the ensemble mean:

$$\bar{f}\left( {x_{i} } \right) = \frac{1}{m}\sum \: _{{run = 1}}^{m} f_{{run}} \left( {x_{i} } \right)$$

and the standard deviation for $\:\theta\:\left(x\right)$, $\nu\:\:\left(x\right)$, and $\:n\left(x\right).\:$These yield both the mean stellar structure and a statistical measure of numerical uncertainty.

The detailed computational sequence is provided in Appendix B.

### Comparison with Runge–Kutta and convergence

To validate the MC solver, we compare its results against a fourth-order Runge–Kutta (RK4) reference solution with step size $\:\varDelta\:\:x\:=\:0.001$. Table 1 shows the first zero $\:{x}_{s}$ for a uniform polytrope ($\:{n}_{c}=\:{n}_{e}=\:2$) across $\sigma\:\:=\:0$ to $\:0.5$. The MC solution (averaged over 10 runs) agrees with RK4 to within $\:0.025-0.066$, with the absolute error increasing with σ due to enhanced stiffness. The standard deviation across MC runs (typically $\:0.01-0.02$) provides a natural uncertainty estimate. Convergence tests (Appendix B) confirm that increasing the number of samples $\:N$ reduces the error as $\:O(1/\sqrt{N}).$

Although the Monte Carlo and Runge–Kutta solutions agree closely for fixed parameter sets, their roles are fundamentally different. The Runge–Kutta method provides a single deterministic solution for a given equation of state, whereas the present problem involves a multi-parameter composite model with intrinsic degeneracies. In such cases, a single trajectory is insufficient to capture the range of physically admissible solutions.

The Monte Carlo framework, by contrast, generates an ensemble of solutions across the parameter space, enabling simultaneous integration and statistical inference. This allows us to quantify uncertainties in the stellar structure and to explore how variations in model parameters affect observable quantities such as mass and radius. Therefore, the advantage of the Monte Carlo method in this work lies not in replacing deterministic solvers, but in extending them to a probabilistic framework suitable for complex, parameter-dependent compact star models.

## Physical analysis of composite polytropic models

### Emden and mass functions

We present the results of MC calculations for composite polytropes, providing a comprehensive depiction of the behavior of both the Emden and the mass functions under various physical scenarios, including Newtonian ($\:\sigma\:=0$), relativistic uniform ($\:{n}_{c}={n}_{e}$), and relativistic composite polytropic models$\:\:{n}_{c}\ne\:{n}_{e}$. In the composite polytrope models studied via repeated MC runs, the central density (ρc), core size parameter ($\:{x}_{c})$, transition width (ε), relativistic parameter (σ), core polytropic index $\:{n}_{\mathrm{c}}$, and envelope polytropic index $\:{n}_{e}$ were systematically varied to probe their impact on both the Emden and mass functions under Newtonian, relativistic, and composite configurations.

The solutions were started at the initial values $\:x=0$, $\:\theta\:\left(0\right)=1$, and $\:\upsilon\:\left(0\right)=0$. We proceed using a finite step size $\:{\Delta\:}x=0.01$ and $\:N\:=\:{2\times\:10}^{6}$ MC samples per integration step, with $\:m\:=\:10$ independent runs for each parameter combination. By using the two relations, $\:{{\upeta\:}}_{0}=\left({n}_{\mathrm{c}}+{n}_{\mathrm{e}}\right)/2$ and $\:{{\upeta\:}}_{1}={n}_{\mathrm{c}}-{{\upeta\:}}_{0}$, we may ascertain the proper values of the constants $\:{{\upeta\:}}_{0}$ and $\:{{\upeta\:}}_{1}$ in Eq. (15). In this case, the polytropic index $\:\mathrm{n}$ may take any value between that of the polytropic indices $\:{n}_{\mathrm{c}}$ and $\:{n}_{\mathrm{e}}$, where $\:{n}_{\mathrm{c}}$ covers the area between the star’s center and the transition zone’s first boundary, and $\:{n}_{\mathrm{e}}$ covers the region between the transition region’s second boundary and the star’s surface.

**Fig. 1:**
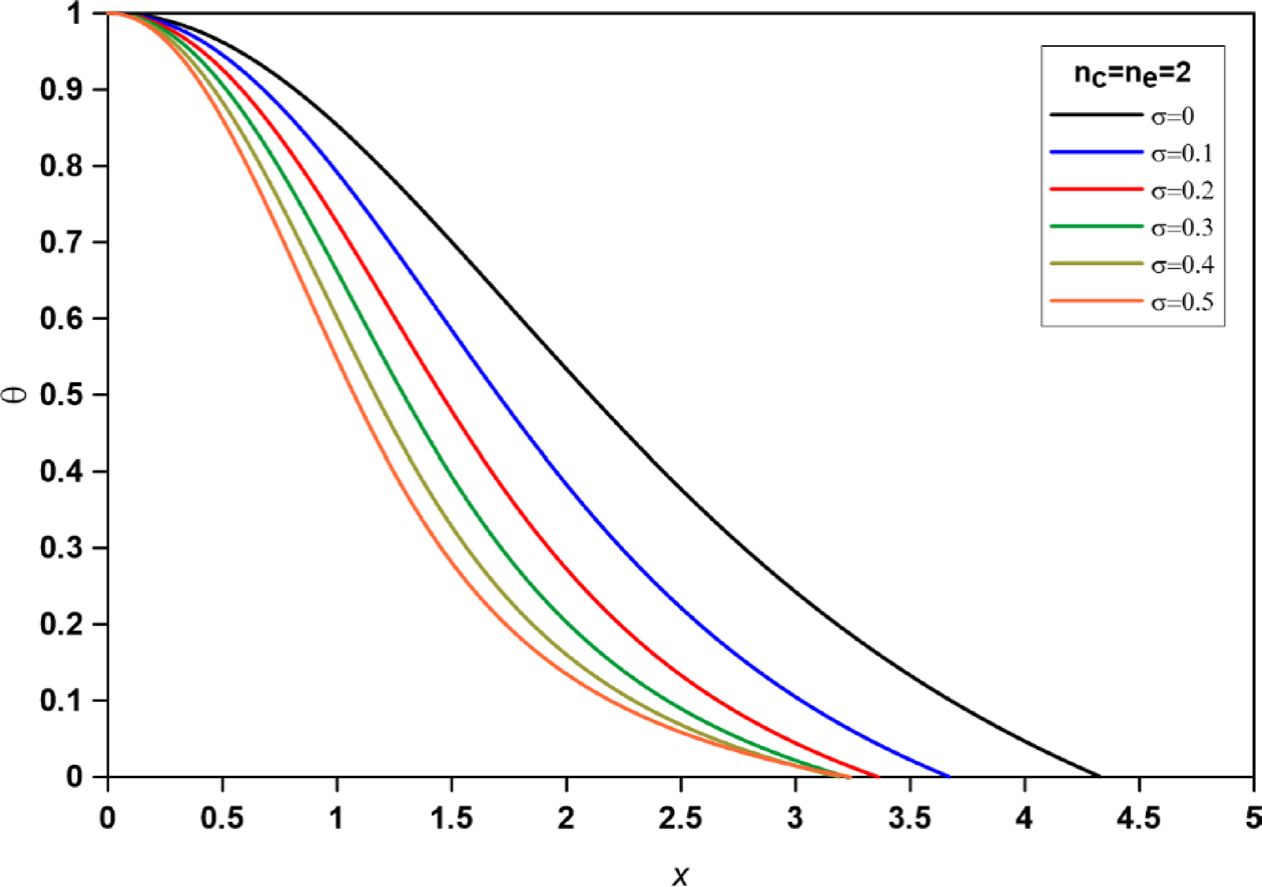
Emden function θ(*x*) profiles for a uniform-index polytrope (n_c_ = n_e_ = 2) under varying relativistic parameter σ, ranging from 0 (Newtonian) to 0.5 (strongly relativistic). Solutions obtained via Monte Carlo integration with *N* = 2 × 10^6^ samples per step over 10 independent runs. Higher σ results in a more rapid decline in θ, indicating enhanced relativistic compression and more compact stellar configurations.

The determination of the Emden function’s first zero, $\:{x}_{s}$, serves as a fundamental diagnostic in polytropic stellar modeling, as it defines the stellar surface radius for a given central density and equation of state. By systematically altering the relativistic parameter σ, we quantify how strong-field gravity modifies the equilibrium structure. Figure 1 distinctly illustrates this influence on a uniform-index polytrope (n_c_ = n_e_= 2), showing that as σ escalates from 0 to 0.5, the Emden function θ declines more rapidly with radius $\:x$, indicating enhanced relativistic compression and a more compact stellar configuration, for instance, at $\:x=2.0$, θ drops from 0.60 (Newtonian) to 0.15 ($\:\sigma\:=0.5$). Table 1 quantitatively supports this trend by comparing $\:{x}_{s}$ calculated via the MC method (averaged over 10 runs, with $\rho \:_{c} = 10^{{15}}$ g cm^-3^, $\:{x}_{c}=\:0.2$, $\:\epsilon\:\:=\:0.01$) with a Runge–Kutta (RK) reference solution as σ increases. The MC results consistently underestimate $\:{x}_{s}$, with the absolute error expanding from 0.025 at $\:\sigma\:=0$ to 0.066 at $\:\sigma\:=0.5$, demonstrating that relativistic effects compound the numerical discrepancy between stochastic and deterministic techniques. This systematic tendency underscores the susceptibility of compact stellar models to σ and the necessity of carefully calibrating MC approaches in strongly relativistic regimes, which is essential for accurately modeling dense astrophysical objects such as neutron stars and white dwarfs.

**Table 1:** The first zero of the Emden function for a polytrope with$\:\:{n}_{c}=\:{n}_{e}=2$, $\:{\rho\:}_{c}={10}^{15}$ g cm^−3^, $\:{x}_{c}$= 0.2, ε = 0.01; calculated for different values of σ.

σ	$\:{x}_{s}$(MC mean)	$\:{x}_{s}$(RK)	Abs. error
0	4.325 ± 0.012	4.350	0.025
0.1	3.666 ± 0.014	3.698	0.032
0.2	3.356 ± 0.016	3.398	0.042
0.3	3.220 ± 0.015	3.271	0.051
0.4	3.186 ± 0.018	3.247	0.061
0.5	3.230 ± 0.019	3.296	0.066

**Fig. 2:**
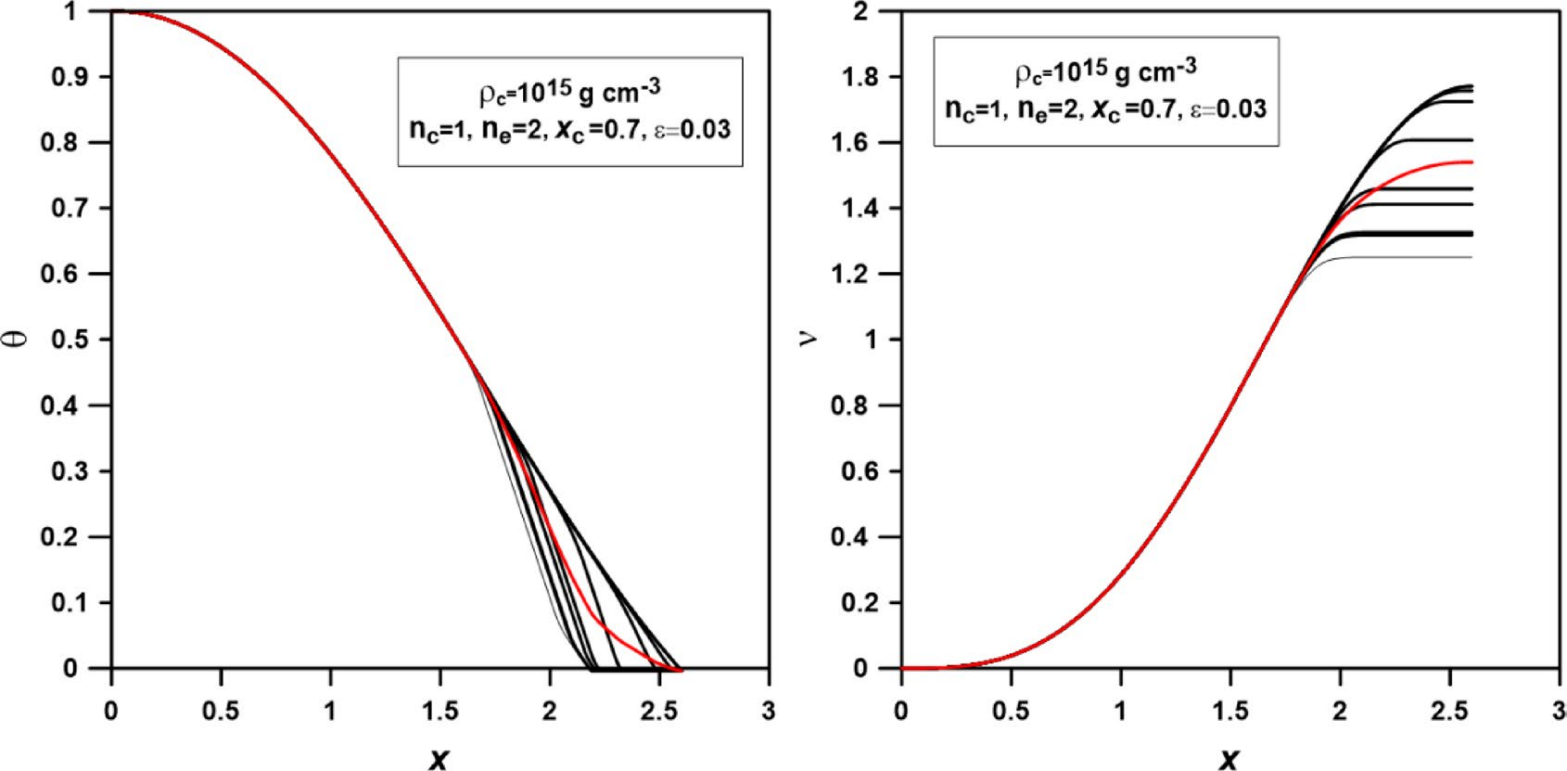
Ensemble solutions for Emden function θ(x) (left) and dimensionless mass function ν(x) (right) over 10 independent Monte Carlo runs (black curves) with ensemble mean (red). Parameters n_c_ =1, n_e_ = 2, ρ_c_ = 10^15^ g cm^− 3^, $\:{x}_{c}$= 0.7, ε = 0.01, and $\sigma\:\:=\:0.1$.

Figure 2 shows ensemble solutions for n_c_ =1, n_e_ = 2, ρ_c_ = 10^15^ g cm^− 3^, $\:{x}_{c}$= 0.7, ε = 0.03, and $\sigma\:\:=\:0.1$. The stochastic MC solver produces a clustered family of solutions for both the Emden function θ(*x*) and the mass function ν(*x*), with a well-behaved representative profile from the ensemble mean. The θ panel shows that the algorithm accurately reproduces the regular central behaviour and decline of θ with minimal stochastic noise in the inner regions. The black curves from 10 independent runs are nearly identical near the centre and remain close throughout the radius. The visible spread between runs is only noticeable near the stellar surface, where θ approaches zero. This creates an uncertainty band near the first zero ($\:{x}_{s}$) and the outermost part of the density profile. Interpolating all trajectories onto a common grid and averaging yields a smooth, monotonic red mean curve without artificial oscillations, supporting the mean model approach to summarise stochastic solutions physically. The behavior of the mass function is similar but more informative for global quantities. The ν(*x*) curves are nearly indistinguishable in the core, indicating a robust enclosed mass that is insensitive to stochastic perturbations. The spread increases with x, indicating a saturating profile. The black curves at large *x* show run-to-run variability in both the total dimensionless mass ν ($\:{x}_{s}$) and the precise radius where mass accumulation stops. This is consistent with the systematic offsets and errors quantified in Table 1 when comparing MC to Runge-Kutta and exploring different σ and ε (Tables 2 and 3). For a fixed parameter set, the red ν(*x*) means curve provides a smooth, monotonic increase that asymptotically levels off, defining mean stellar mass and supporting the use of ensemble-averaged profiles and standard deviations for diagnostic purposes.

**Fig. 3:**
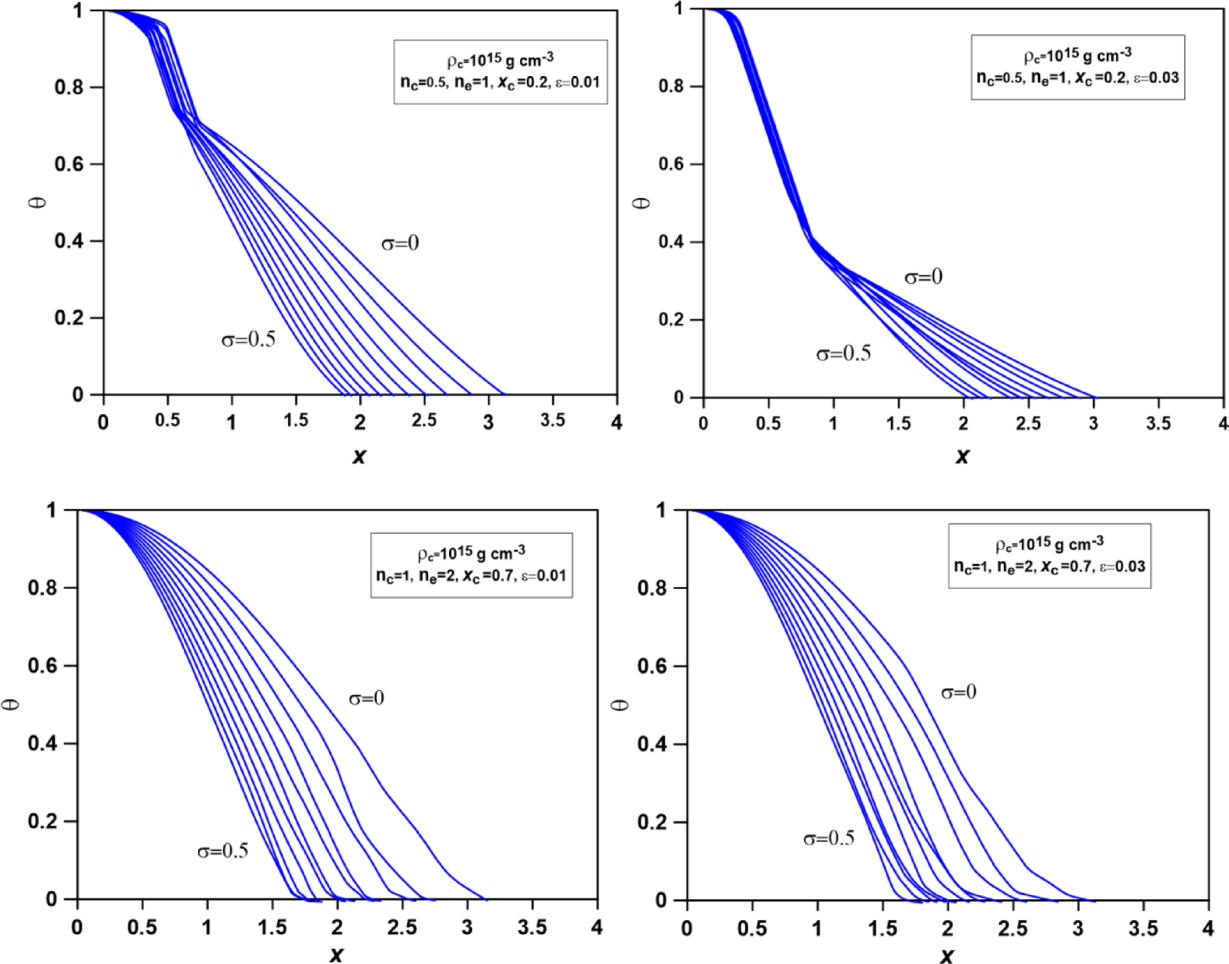
The Emden function of the composite polytrope with central density ρc = 10^15^: Top panel for n_c_=0.5, n_e_=1, $\:{x}_{\mathrm{c}}$=0.2; ε = 0.01, 0.03, bottom panel for n_c_=1, n_e_=2, $\:{x}_{\mathrm{c}}$=0.7; ε = 0.01, 0.03.

**Fig. 4:**
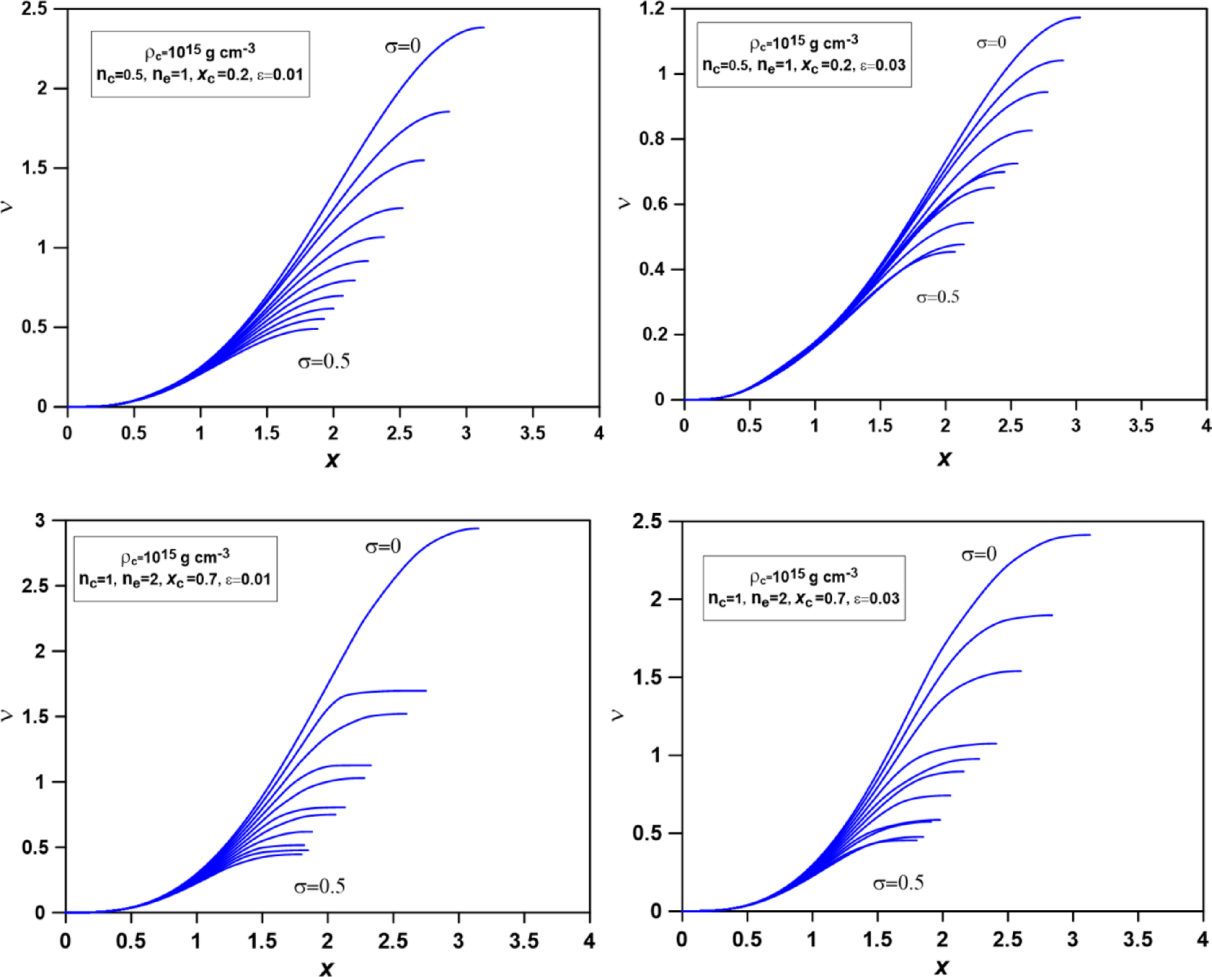
The mass function of the composite polytrope with central density ρ_c_ = 10^15^: Top panel for *n*_*c*_=0.5, *n*_*e*_=1, $\:{x}_{\mathrm{c}}$=0.2; ε = 0.01, 0.03; bottom panel for *n*_*c*_=1, *n*_*e*_=2, $\:{x}_{\mathrm{c}}$=0.7; ε = 0.01, 0.03.

We examine the structural properties of two distinct composite polytropic models under varying relativistic parameters (σ) and transition widths (ε). Model 1 features a softer composition with $\:{n}_{c}=0.5$, $\:{n}_{e}=1$, and $\:{x}_{c}=0.2$, while Model 2 represents a stiffer configuration with $\:{n}_{c}=1$, $\:{n}_{e}=2$, and $\:{x}_{c}=0.7$. Both models are computed with central density $\rho \:_{c} = 10^{{15}}$ g cm_-3_ and transition widths $\:\varepsilon\:\:=\:0.01$ and $\:0.03$ (Figs. 3 and 4), allowing for a comprehensive comparison of how internal stratification and relativistic gravity jointly determine stellar equilibrium.

The data in Tables 2 and 3 reveal systematic trends in the Emden radius $\:\left({x}_{s}\right)$, dimensionless mass ν ($\:{x}_{s}$), and derived compactness parameters as functions of σ and ε. For both models, increasing σ from 0 to 0.5 leads to significant reductions in both $\:{x}_{s}$ and ν ($\:{x}_{s}$). In Model 1 with $\:\varepsilon\:=0.01$, $\:{x}_{s}$ contracts from 3.14 to 1.859 (40% reduction) while ν ($\:{x}_{s}$) decreases from 2.3734 to 0.497 (79% reduction). Model 2 shows even more dramatic effects: with $\:\varepsilon\:=0.01$, $\:{x}_{s}$ reduces from 3.074 to 1.7657 (44% reduction) and ν ($\:{x}_{s}$) plummets from 2.558 to 0.4567 (85% reduction). The transition width ε significantly modifies these responses. For Model 1 at $\:\sigma\:=0$, a sharper transition ($\:\varepsilon\:=0.01$) yields $\:{x}_{s}=3.14$ and $\:\nu\:=2.3734$, while a smoother transition ($\:\varepsilon\:=0.03$) produces a smaller configuration with $\:{x}_{s}=3.016$ and $\:\nu\:=1.1466$. Similarly, for Model 2 at $\:\sigma\:=0$, $\:\varepsilon\:=0.01$ gives $\:{x}_{s}=3.074\:$ and $\:\nu\:=2.558\:$, while $\:\varepsilon\:=0.03$ yields $\:{x}_{s}=3.0522$ and $\:\nu\:=2.6073$. The compactness ratio ν($\:{x}_{s}$)/$\:{x}_{s}$ systematically decreases with increasing σ for both models and both ε values, though the rate of decrease varies with model stiffness and transition sharpness. The radial profiles of θ(*x*) and ν(*x*) provide visual confirmation and additional insights beyond the tabulated boundary values. For both models, θ(*x*) declines more steeply with increasing σ, with smoother transitions (ε = 0.03) producing more gradual descents compared to sharper transitions (ε = 0.01). This difference is particularly pronounced near the core boundary parameter $\:{r}_{c}$, where sharper transitions create more abrupt changes in slope. Model 2, with its larger core ($\:{x}_{c}$=0.7) and stiffer indices, shows more distinct structural breaks than Model 1. The mass functions ν(*x*) demonstrate similar systematic behavior: saturation occurs earlier and at lower values for higher σ. Models with smoother transitions exhibit more extended mass accumulation, while sharper transitions lead to a more rapid approach to asymptotic mass values. The influence of core size is evident in the different growth patterns: Model 2’s larger core produces a steeper initial rise in ν(*x*) within the core region, followed by a more gradual increase in the envelope compared to Model 1’s more uniform mass distribution.

These results collectively demonstrate that both relativistic compactness and internal compositional structure critically determine equilibrium configurations of layered compact objects. The more dramatic response of Model 2 to increasing σ suggests that stiffer, more massive cores undergo particularly efficient gravitational collapse, potentially explaining observed mass-radius relationships for neutron stars with different internal compositions. The sensitivity to transition width ε indicates that the sharpness of core-envelope interfaces significantly affects global properties, especially in non-relativistic regimes. For astrophysical applications, particularly in modeling neutron stars and massive white dwarfs with stratified interiors, these findings underscore the need to incorporate both variable polytropic indices and realistic transition profiles. The composite polytropic approach, when solved with appropriate numerical methods accounting for relativistic effects, provides a robust framework for interpreting observational data and constraining the equation of state in dense stellar interiors. The quantitative relationships established here between model parameters (*n*_*c*_, *n*_*e*_, $\:{x}_{c}$, ε, σ) and structural outcomes ($\:{x}_{s}$, ν($\:{x}_{s}$), compactness) provide valuable constraints for theoretical models of compact objects and highlight the complex interplay between microphysical properties (encoded in polytropic indices) and macroscopic gravitational effects in determining stellar structure.

**Table 2:** The first zero of the Emden function ($\:{x}_{s}$), the mass function at $\:{x}_{s}$ for a polytrope with n_c_=0.5, n_e_=1, $\:{x}_{c}$= 0.2, ε = 0.01, and 0.03 (Model 1).

σ	$\:{x}_{s}$	${\upsilon }\left( {x_{s} } \right)$	${{\upsilon \left( {x_{s} } \right)} \mathord{\left/ {\vphantom {{\upsilon \left( {x_{s} } \right)} {x_{s} }}} \right. \kern-\nulldelimiterspace} {x_{s} }}$	${{\upsilon \left( {x_{s} } \right)} \mathord{\left/ {\vphantom {{\upsilon \left( {x_{s} } \right)} {\upsilon \left( {x_{s} } \right)_{{\sigma = 0}} }}} \right. \kern-\nulldelimiterspace} {\upsilon \left( {x_{s} } \right)_{{\sigma = 0}} }}$	$\:{x}_{s}$	${\upsilon }\left( {x_{s} } \right)$	${{\upsilon \left( {x_{s} } \right)} \mathord{\left/ {\vphantom {{\upsilon \left( {x_{s} } \right)} {x_{s} }}} \right. \kern-\nulldelimiterspace} {x_{s} }}$	${{\upsilon \left( {x_{s} } \right)} \mathord{\left/ {\vphantom {{\upsilon \left( {x_{s} } \right)} {\upsilon \left( {x_{s} } \right)_{{\sigma = 0}} }}} \right. \kern-\nulldelimiterspace} {\upsilon \left( {x_{s} } \right)_{{\sigma = 0}} }}$
	ε = 0.01				ε = 0.03			
0	3.14	2.373 ± 0.005	0.756	1	3.016	1.146 ± 0.026	0.380	1
0.05	2.871	1.855 ± 0.006	0.646	0.782	2.89	1.045 ± 0.045	0.362	0.911
0.1	2.669	1.518 ± 0.028	0.569	0.640	2.765	0.944 ± 0.048	0.341	0.823
0.15	2.505	1.271 ± 0.013	0.507	0.536	2.641	0.826 ± 0.046	0.313	0.72
0.2	2.368	1.052 ± 0.006	0.445	0.444	2.537	0.751 ± 0.048	0.296	0.655
0.25	2.249	0.915 ± 0.003	0.407	0.386	2.437	0.696 ± 0.063	0.286	0.607
0.3	2.149	0.793 ± 0.002	0.369	0.334	2.349	0.623 ± 0.048	0.265	0.543
0.35	2.061	0.694 ± 0.007	0.337	0.292	2.267	0.585 ± 0.036	0.258	0.51
0.4	1.990	0.613 ± 0.007	0.308	0.258	2.195	0.545 ± 0.019	0.248	0.475
0.45	1.920	0.553 ± 0.000	0.288	0.233	2.121	0.497 ± 0.024	0.234	0.433
0.5	1.859	0.497 ± 0.005	0.267	0.209	2.059	0.445 ± 0.023	0.216	0.388

**Table 3:** The first zero of the Emden function ($\:{x}_{s}$), the mass function at $\:{x}_{s}$ for a polytrope with *n*_*c*_=1, *n*_*e*_=2, $\:{x}_{c}$= 0.7, ε = 0.01, and 0.03 (Model 2).

σ	$\:{x}_{s}$	${\upsilon }\left( {x_{s} } \right)$	${{\upsilon \left( {x_{s} } \right)} \mathord{\left/ {\vphantom {{\upsilon \left( {x_{s} } \right)} {x_{s} }}} \right. \kern-\nulldelimiterspace} {x_{s} }}$	${{\upsilon \left( {x_{s} } \right)} \mathord{\left/ {\vphantom {{\upsilon \left( {x_{s} } \right)} {\upsilon \left( {x_{s} } \right)_{{\sigma = 0}} }}} \right. \kern-\nulldelimiterspace} {\upsilon \left( {x_{s} } \right)_{{\sigma = 0}} }}$	$\:{x}_{s}$	${\upsilon }\left( {x_{s} } \right)$	${{\upsilon \left( {x_{s} } \right)} \mathord{\left/ {\vphantom {{\upsilon \left( {x_{s} } \right)} {x_{s} }}} \right. \kern-\nulldelimiterspace} {x_{s} }}$	${{\upsilon \left( {x_{s} } \right)} \mathord{\left/ {\vphantom {{\upsilon \left( {x_{s} } \right)} {\upsilon \left( {x_{s} } \right)_{{\sigma = 0}} }}} \right. \kern-\nulldelimiterspace} {\upsilon \left( {x_{s} } \right)_{{\sigma = 0}} }}$
	ε = 0.01				ε = 0.03			
0	3.074	2.558 ± 0.043	0.832	1	3.052	2.607 ± 0.055	0.854	1
0.05	2.737	2.030 ± 0.019	0.742	0.794	2.783	1.963 ± 0.029	0.705	0.753
0.1	2.459	1.443 ± 0.016	0.587	0.564	2.555	1.433 ± 0.029	0.561	0.550
0.15	2.371	1.267 ± 0.012	0.535	0.496	2.349	1.208 ± 0.018	0.514	0.463
0.2	2.156	1.028 ± 0.096	0.477	0.402	2.254	1.051 ± 0.014	0.466	0.403
0.25	2.112	0.852 ± 0.011	0.404	0.333	2.125	0.823 ± 0.015	0.388	0.316
0.3	2.038	0.726 ± 0.011	0.356	0.284	2.006	0.691 ± 0.012	0.345	0.265
0.35	1.914	0.622 ± 0.069	0.325	0.244	1.958	0.645 ± 0.010	0.330	0.248
0.4	1.870	0.535 ± 0.077	0.287	0.209	1.867	0.565 ± 0.073	0.303	0.217
0.45	1.798	0.502 ± 0.059	0.280	0.197	1.807	0.460 ± 0.078	0.255	0.177
0.5	1.765	0.456 ± 0.064	0.259	0.179	1.758	0.440 ± 0.065	0.25	0.169

The ensemble standard deviations reported in Tables 2 and 3 quantify the numerical reproducibility of the MC solver across m = 10 independent runs ($\:N=2\times\:{10}^{6}$ samples/step). For Model 1 at σ = 0, ε = 0.01, $\:{x}_{s}=3.14\pm\:0.005$ represents a relative uncertainty $\:\varDelta\:{x}_{s}/{x}_{s}=0.16$, which propagates to $\:\varDelta\:R\:\approx\:\:\pm\:0.19$ km assuming the characteristic length scale L ≈ 12 km (Eq. 25). Similarly, $\:\nu\:\left({x}_{s}\right)=2.373\pm\:0.005$ yields $\:\varDelta\:M\approx\:\pm\:0.008{M}_{\odot\:}$ (< 0.2% relative error) through $\:{M}_{0}\approx\:1.6{M}_{\odot\:}$ (Eq. 26). These uncertainties are ~ 60× smaller than NICER radius precision (± 0.3 km) and ~ 5× smaller than GW170817 mass constraints ($\:\pm\:0.04{M}_{\odot\:}$), confirming that the reported SD reflects stochastic sampling noise (O(1/√N) scaling, Appendix B) rather than astrophysical EoS variability. Surface uncertainties grow modestly with σ due to enhanced numerical stiffness near θ→0, but remain below 0.3% relative across all configurations, validating the MC method’s robustness for CTOV integration of composite polytropes.

For the composite polytropic model with $\:{n}_{c}=1$, $\:{n}_{e}=2$, $\:{x}_{c}=0.7$, $\:\varepsilon\:=0.01$, the dimensionless mass $\:\nu\:\left({x}_{s}\right)\:$and the first zero of the Emden function $\:{x}_{s}\:$ provides the key quantities needed to reconstruct the neutron star’s physical mass and radius through the scaling relations introduced in Eqs. (24)–(26) and the boundary conditions of the CTOV system. Table 4 summarizes the mass-radius relation generated by varying $\:\sigma\:\:$from 0.05 to 0.5: as $\:\sigma\:\:$ increases, $\:{x}_{s}\:$decreases from 2.737 to 1.765 while $\:\nu\:\left({x}_{s}\right)\:$decreases from 2.030 ± 0.019 to 0.456 ± 0.064, yielding physical masses from 0.5191 $\:{M}_{\odot\:}\:$to 3.6879 $\:{M}_{\odot\:}\:$and radii from 9.3968 km to 19.1625 km. These results demonstrate the model’s ability to produce realistic neutron star configurations spanning the observed mass range while maintaining the stratified core-envelope structure characteristic of composite polytropes. More extensive calculations of masses and radii across additional parameter combinations, along with comparisons with observational data, will be presented in "Mass-Radius Relation and comparison with observations" and "The maximum mass of neutron stars".

**Table 4:** Mass-radius relation for model with *n*_*c*_=1, *n*_*e*_=2, $\:{x}_{c}$= 0.7, ε = 0.01.

σ	$\:{x}_{s}$	${\upsilon }\left( {x_{s} } \right)$)	M (M$_{ \odot }$)	*R* (km)
0.05	2.737	2.030 ± 0.019	$\:0.5191$	$\:9.3968$
0.1	2.459	1.443 ± 0.016	$\:1.0438$	$\:11.9394$
0.15	2.371	1.267 ± 0.012	$\:1.6837$	$\:14.0994$
0.2	2.156	1.028 ± 0.096	$\:2.1033$	$\:14.8043$
0.25	2.112	0.852 ± 0.011	$\:2.4362$	$\:16.2139$
0.3	2.038	0.726 ± 0.011	$\:2.7288$	$\:17.1391$
0.35	1.914	0.622 ± 0.069	$\:2.9651$	$\:17.3860$
0.4	1.870	0.535 ± 0.077	$\:3.0960$	$\:18.1591$
0.45	1.798	0.502 ± 0.059	$\:3.4664$	$\:18.5191$
0.5	1.765	0.456 ± 0.064	$\:3.6879$	$\:19.1625$

## Stability analysis

Building directly on the density, pressure, and mass profiles established in Sect.  4 (Figs. 1, 2, 3 and 4; Tables 2 and 3), we now verify that the composite polytropic configurations satisfy the fundamental physical and dynamical stability criteria required for realistic neutron star models. To investigate the stability of compact star models, we apply a set of fundamental physical and dynamical conditions^44,45^. These criteria ensure that the composite polytropic configurations are not only mathematically valid but also physically realisable and stable against perturbations. The stability analysis is intrinsically linked to the mass-radius relation through several key physical mechanisms, which we detail below^46^.

### Basic physical requirements

First, the density $\:\rho\:\left(x\right)$ and pressure $\:P\left(x\right)\:$must be positive, finite, and free from singularities within the stellar interior. That is:

30 $$\rho \:\left( x \right) \ge \:0,P\left( x \right) \ge \:0,$$

for all x from 0 to x_s.

For composite polytropic models, the density and pressure are given by:

31 $$\rho \:\left( x \right) = \rho \:_{c} \theta \:\left( x \right)^{{n\left( x \right)}} ,P\left( x \right) = K\rho \:_{c}^{{1 + 1/n\left( x \right)}} \theta \:\left( x \right)^{{n\left( x \right) + 1}} .$$

**Fig. 5:**
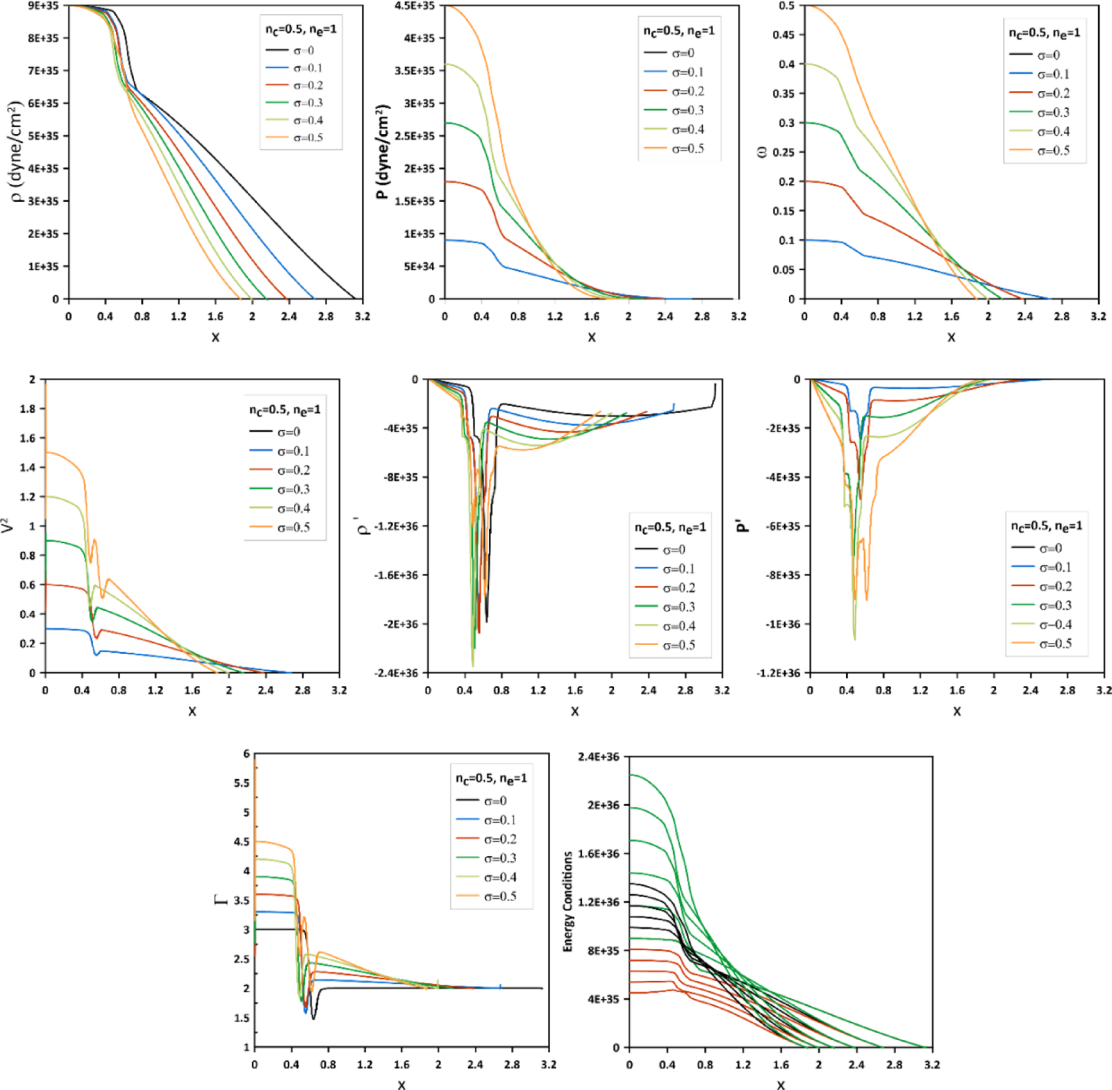
Stability diagnostics for the composite polytropic model with $\:{n}_{c}=0.5$, $\:{n}_{e}=1$, $\:{x}_{c}=0.2$, $\:{\rho\:}_{c}={10}^{15}\mathrm{g}\mathrm{c}{\mathrm{m}}^{-3}$, and $\:\varepsilon\:=0.01$, evaluated for $\:\sigma\:=0-0.5$. The figure shows the monotonic behavior of the density and pressure profiles, the adiabatic index $\:{\Gamma\:}\left(x\right)$, and the energy conditions, confirming hydrostatic and dynamical stability in the allowed parameter range. Sound speed remains subluminal ($\:{v}_{s}<c$) for σ ≤ 0.3; superluminal instability develops for σ > 0.3.

**Fig. 6:**
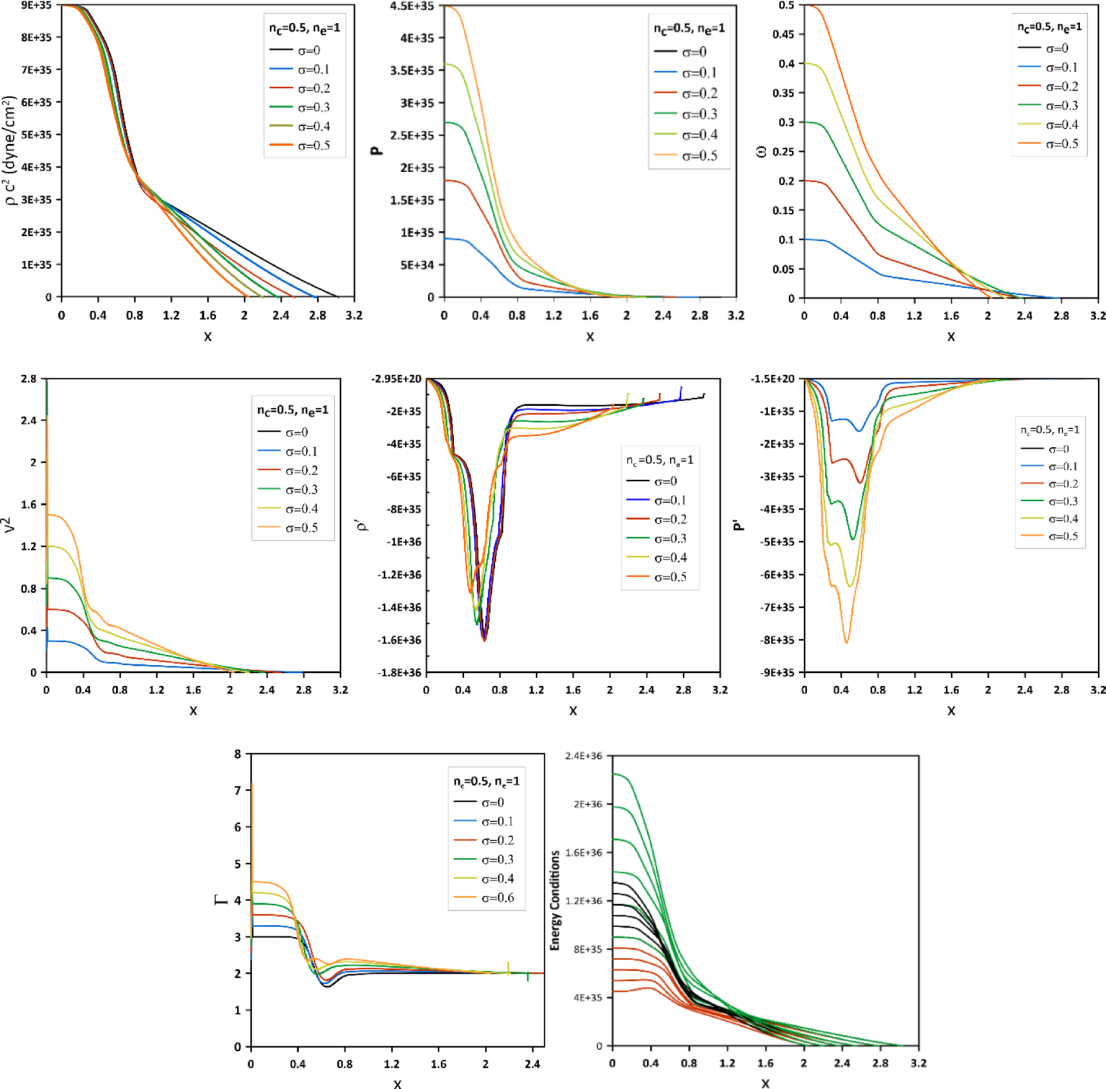
Stability diagnostics for the composite polytropic model with $\:{n}_{c}=0.5$, $\:{n}_{e}=1$, $\:{x}_{c}=0.2$, $\:{\rho\:}_{c}={10}^{15}\mathrm{g}\mathrm{c}{\mathrm{m}}^{-3}$, and $\:\varepsilon\:=0.03$, evaluated for $\:\sigma\:=0-0.5$. The figure shows the monotonic behavior of the density and pressure profiles, the adiabatic index $\:{\Gamma\:}\left(x\right)$, and the energy conditions, confirming hydrostatic and dynamical stability in the allowed parameter range. Subluminal sound speeds for σ ≤ 0.3; causality violation ($\:{v}_{s}>c$) for σ > 0.3 indicates physical instability.

**Fig. 7:**
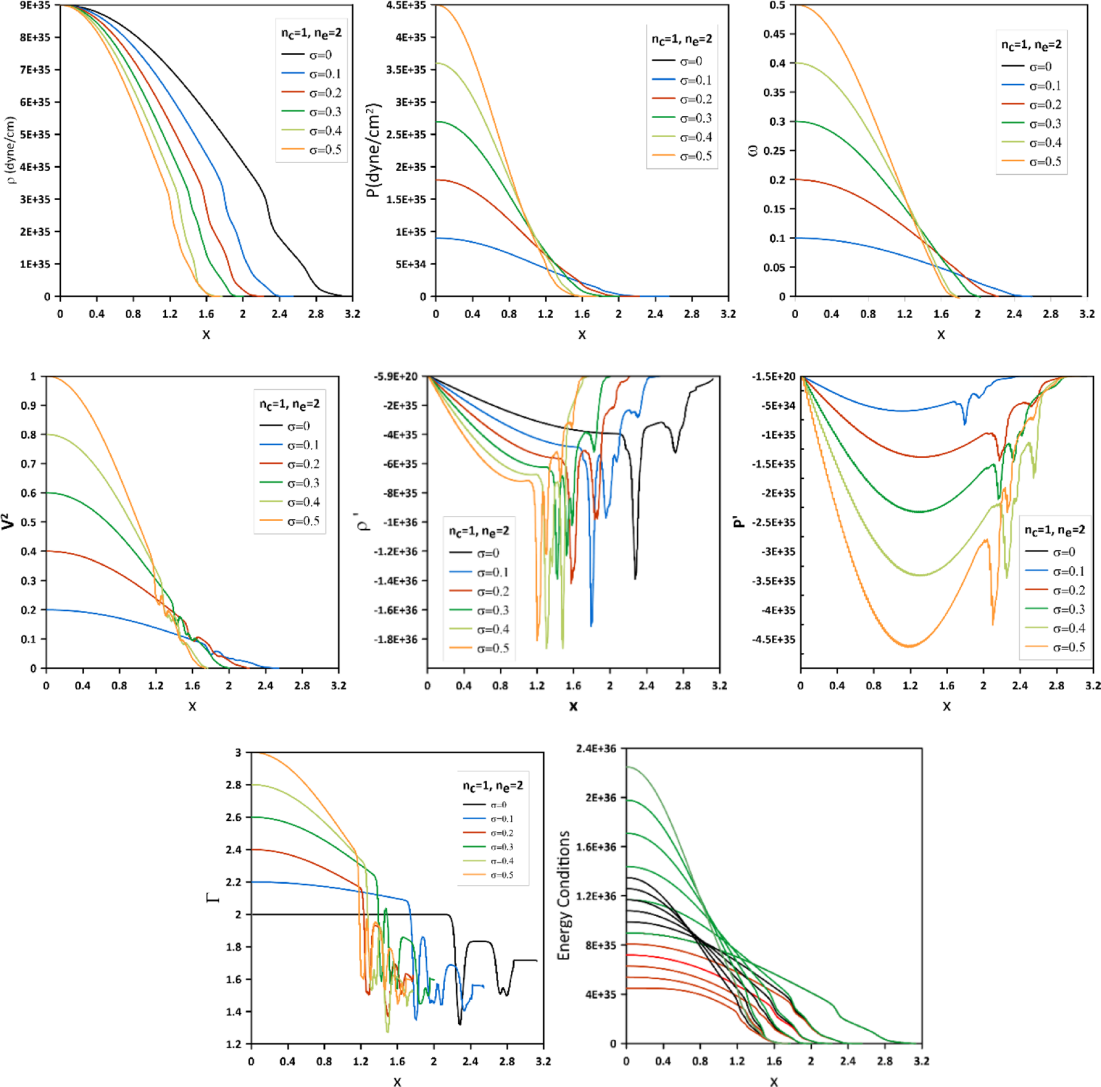
Stability diagnostics for the composite polytropic model with $\:{n}_{c}=1$, $\:{n}_{e}=2$, $\:{x}_{c}=0.7$, $\:{\rho\:}_{c}={10}^{15}\mathrm{g}\mathrm{c}{\mathrm{m}}^{-3}$, and $\:\varepsilon\:=0.01$, evaluated for $\:\sigma\:=0-0.5$. The figure shows the monotonic behavior of the density and pressure profiles, the adiabatic index $\:{\Gamma\:}\left(x\right)$, sound speed, and the energy conditions, confirming hydrostatic and dynamical stability in the allowed parameter range.

**Fig. 8:**
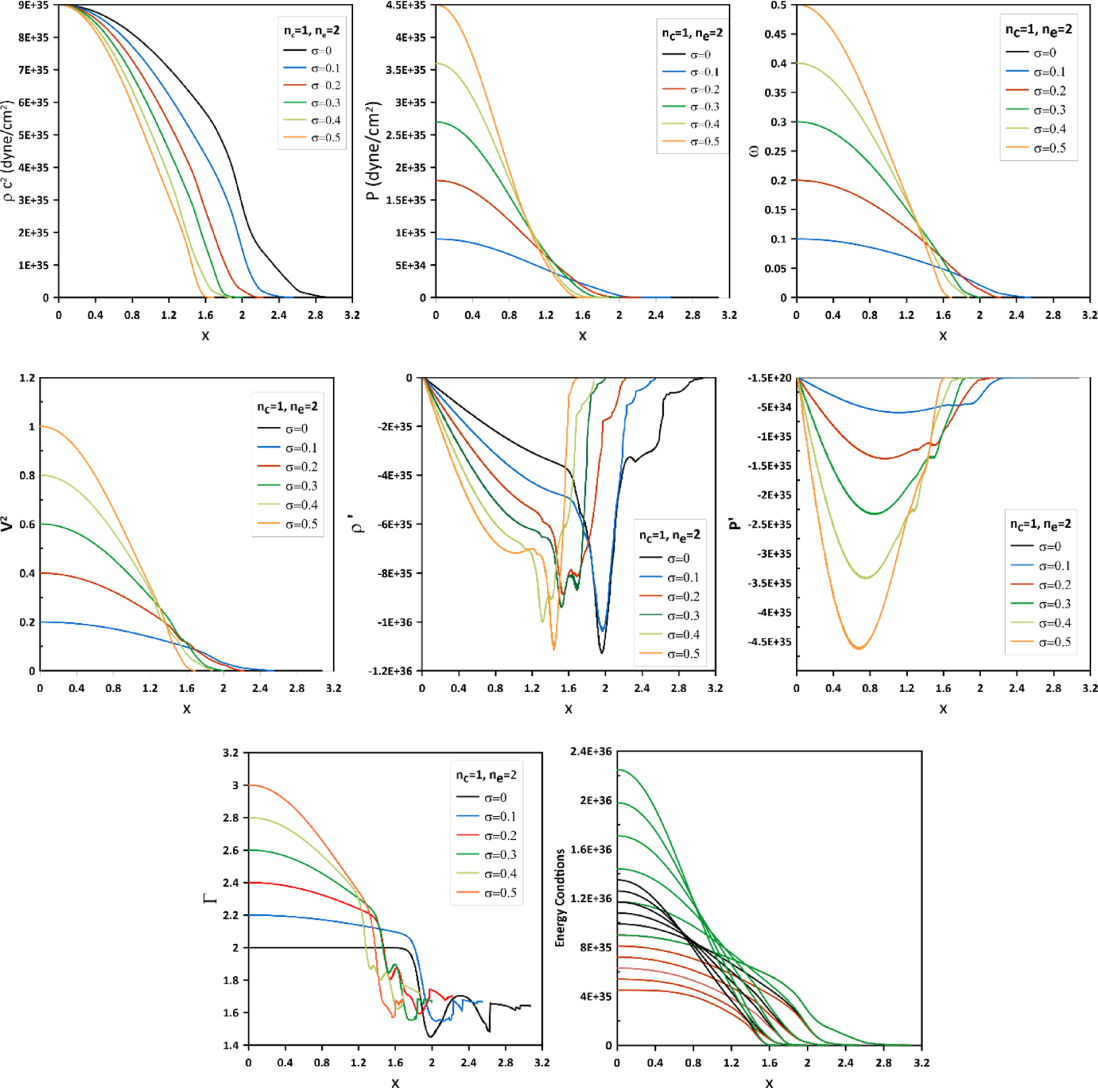
Stability diagnostics for the composite polytropic model with $\:{n}_{c}=1$, $\:{n}_{e}=2$, $\:{x}_{c}=0.7$, $\:{\rho\:}_{c}={10}^{15}\mathrm{g}\mathrm{c}{\mathrm{m}}^{-3}$, and $\:\varepsilon\:=0.03$, evaluated for $\:\sigma\:=0-0.5$. The figure shows the monotonic behavior of the density and pressure profiles, the adiabatic index $\:{\Gamma\:}\left(x\right)$, sound speed, and the energy conditions, confirming hydrostatic and dynamical stability in the allowed parameter range.

These conditions are satisfied everywhere, as confirmed by the monotonic and regular profiles shown in Figs. 5, 6, 7 and 8. Both density and pressure decrease smoothly from their central maximum values to zero at the surface.

Second, the density and pressure gradients must be negative throughout the interior:

$\:\frac{d\rho\:}{dx}<0,\frac{dP}{dx}<0,\:$ for all $\:x$ from 0 to $\:{x}_{\mathrm{s}}$.

This ensures hydrostatic equilibrium and prevents unphysical inversions where density or pressure would increase outward. Figures 5, 6, 7 and 8 confirm that both gradients remain negative for all models. Additionally, the pressure must vanish at the surface: $\:P\left({x}_{\mathrm{s}}\right)=0$, which is satisfied by the smoothly decreasing profiles.

### Adiabatic index and dynamical stability

For an isotropic fluid sphere, the adiabatic index $\:{\Gamma\:}\left(x\right)$ governs dynamical stability against radial perturbations. It is defined as:

32 $$\:\Gamma \left( x \right) = \frac{{\rho + P}}{P}\frac{{dP}}{{d\rho }}.$$

The condition for stability against radial oscillations is that $\:{\Gamma\:}\left(x\right)$ must be greater than 4/3 throughout the stellar interior^47^. If $\:{\Gamma\:}\left(x\right)$ falls below 4/3 in any region, the star becomes unstable to collapse. Figures 5, 6, 7 and 8 show the adiabatic index $\:{\Gamma\:}\left(x\right)$ for all models. For the stiff configuration ($\:{n}_{c}\:=\:1,\:{n}_{e}\:=\:2$), $\:{\Gamma\:}\left(x\right)$ exceeds 4/3 for all $\:\sigma\:$ from 0 to 0.5. The minimum value of $\:{\Gamma\:}\left(x\right)$ occurs near the surface but remains safely above the critical threshold. For the soft configuration ($\:{n}_{c}\:=\:0.5,\:\:{n}_{e}\:=\:1$), $\:{\Gamma\:}\left(x\right)>\frac{4}{3}$ for all σ values.

The adiabatic index condition directly determines the turning-point criterion along a sequence of equilibrium models. For a given equation of state, as the central density $\:{\rho}_{c}$increases, the stellar mass $\:M$ generally rises to a maximum and then declines. The point where $\:dM/d{\rho}_{c}\:=\:0$ marks the onset of radial instability. In our composite polytropes, the condition $\:{\Gamma\:}\left(x\right)>\frac{4}{3}$ ensures that models lie on the stable ascending branch of the mass-radius curve. The steepness of this branch, and hence the maximum attainable mass, depends sensitively on how Gamma varies with radius.

### Causality condition

The speed of sound $\:\left({v}_{s}\right)$ must be less than the speed of light c to maintain causality. The condition is:

$\:0<\frac{{v}_{s}^{2}}{{c}^{2}}=\frac{dP}{d\rho\:}<1,\:\:$throughout the stellar interior.

Figures 5, 6, 7 and 8 display the sound speed squared in units of c. For the stiff configuration ($\:{n}_{c}=1,\:\:{n}_{e}=2$), the causality condition is satisfied throughout the stellar interior for all $\:\sigma\:$ from 0 to 0.5. The sound speed remains below c for all radii. For the soft configuration ($\:{n}_{c}=0.5,\:\:{n}_{e}=1$), we find that at higher relativistic strengths ($\:\sigma\:$ = 0.4 to 0.5), the sound speed exceeds the speed of light in the inner core region. This superluminal behavior signals a breakdown of physical admissibility and indicates that such highly relativistic configurations are not causal. Consequently, for these composite models, the physically acceptable parameter range is restricted to $\:\sigma\:\:\le\:\:0.3$. The violation at large σ reflects the combined effect of strong relativistic compression and the softness of the core equation of state, which drives $\:dP/d\rho$ beyond unity. The causality condition imposes an upper bound on the stiffness of the equation of state and, consequently, on the compactness $\:M/R$. This constraint directly limits how small the radius R can become for a given mass M, thereby shaping the high-compactness end of the mass-radius relation.

### Zeldovich condition

To ensure non-exotic matter, the Zeldovich condition^48^ must hold at the center:

33 $$0 < \omega \:_{c} = \frac{{P_{c} }}{{\rho \:_{c} c^{2} }} = \sigma < 1.$$

This condition is verified for all models in our study, as σ ranges from 0 to 0.5. The Zeldovich condition ensures that the central pressure is physically attainable and that the equation of state does not violate fundamental thermodynamic principles. This restricts the high-density regime of the mass-radius relation: models with σ approaching unity) would approach the causal limit and yield the maximum possible compactness for a given equation of state.

### Energy conditions

The following energy conditions for an isotropic perfect fluid are satisfied throughout the interior (see Figs. 5, 6, 7 and 8):

- Null energy condition (NEC): ($\:\rho\:+\:P\:\ge\:0$).
- Weak energy condition (WEC): ($\:\rho\:\ge\:0),\:(\rho\:+\:P\:\ge\:0$).
- Strong energy condition (SEC): ($\:\rho\:+\:3P\:\ge\:0$).
- Dominant energy condition (DEC): ($\:\rho\:-\:P\:\ge\:0$).

The consistent satisfaction of all energy conditions guarantees that the predicted mass-radius curves correspond to physically realisable stellar structures.

The smooth, differentiable transition in $\:n\left(x\right)$ at the core-envelope interface prevents discontinuities that could trigger shear or interfacial instabilities, while a continuous sound-speed profile avoids wave-steering artifacts. The convexity of the equation of state confirms thermodynamic stability ($\:{d}^{2}P/d{\rho\:}^{2}>0$) and compliance with the Le Châtelier principle. The MC-based integration further substantiates numerical stability, as ensemble-averaged profiles from multiple independent runs exhibit minimal scatter in the core and converge to well-behaved mean solutions. These models collectively produce a stable, physically admissible M–R curve that can be directly compared with astrophysical observations of compact objects. The composite polytropic framework yields stable, causal, and energetically permissible models for stratified compact objects such as neutron stars and high-mass white dwarfs across the studied parameter space, providing a reliable foundation for future extensions, including rotation and more realistic EoS.

## Mass-radius relation and comparison with observations

The mass-radius relation is one of the most important observational diagnostics for neutron stars. Different equations of state predict different relationships between mass and radius, and precise measurements of both quantities can constrain the internal composition of compact objects. In this section, we present the theoretical mass-radius relations derived from our composite polytropic models, compare them with observational data, and provide the maximum mass analysis. Table 5 presents a comprehensive compilation of 21 neutron stars and neutron‑star candidates with reliably measured masses and radii, categorised by observational method. The data span a wide range of $\:\left(0.85\right)$ to $\:(2.35\hspace{0.17em}{M}_{\odot\:})$ and a radius range of approximately $\:\left(7.3\right)$ to $\:(13.0\hspace{0.17em}\mathrm{km})$, corresponding to mean densities $\:\stackrel{-}{{\uprho\:}}\sim\:\left(2.7-9.1\right)\times\:{10}^{14}\hspace{0.17em}{\mathrm{g\:cm}}^{-3}.$ High‑mass X‑ray binaries exhibit masses between $\:0.85$ $\:\mathrm{a}\mathrm{n}\mathrm{d}\:1.49\hspace{0.17em}{M}_{\odot\:}\:$and relatively compact radii of $\:(7.9-9.2\hspace{0.17em}\mathrm{km})$. For instance, Cen X‑3 yields $\:M=1.49\pm\:0.49\hspace{0.17em}{M}_{\odot\:}\:$and $\:R=9.178\pm\:0.13\hspace{0.17em}\mathrm{km}\:\left(\stackrel{-}{{\uprho\:}}\approx\:9.1\times\:{10}^{14}\hspace{0.17em}{\mathrm{g\:cm}}^{-3}\right)$, indicating a dense, compact configuration. Low‑mass X‑ray binaries (LMXBs) split into two subgroups. Thermonuclear burst sources (e.g., 4U 1608‑52: $\:M=1.74\pm\:0.14\hspace{0.17em}{M}_{\odot\:},\:R=9.3\pm\:1.0\hspace{0.17em}\mathrm{km}$; 4U 1820‑30: $\:M\:=\:1.58\:\pm\:0.3\:{M}_{\odot\:},\:\:R\:=\:9.11\:\pm\:0.4\hspace{0.17em}\:km$ favor radii of $\:9-11\hspace{0.17em}\mathrm{km}$ at masses $\:1.4-1.8\hspace{0.17em}{M}_{\odot\:}$, and quiescent LMXBs such as X7 $\:(M\:=\:1.1\:\pm\:0.3\hspace{0.17em}{M}_{\odot\:}$, $\:R\:=\:12\:\pm\:1.0\hspace{0.17em}\:km$) and the globular‑cluster source M13 $\:(M\:=\:1.38\:\pm\:0.2\hspace{0.17em}{M}_{\odot\:}$, $\:R\:=\:9.95\:\pm\:0.28\hspace{0.17em}\mathrm{k}\mathrm{m})$ show larger radii ( $\:\gtrsim\:12\:\mathrm{km}$) at slightly lower masses, suggesting a softer equation of state in these systems. Millisecond pulsars observed with NICER and radio timing provide precise individual measurements. PSR J0030+0451 has $\:M\approx\:1.34\pm\:014,{M}_{\odot\:}$ and $\:R\approx\:12.71\pm\:1.19\:\mathrm{km}$, while PSR J0437-4715 gives $\:M=1.44\pm\:0.07{M}_{\odot\:}$ and $\:R=13.02\pm\:0.27\hspace{0.17em}\mathrm{km}$. Three systems set critical lower bounds on the maximum mass: PSR J0348+0432 $\:(M\:=\:2.01\:\pm\:0.04\hspace{0.17em}{M}_{\odot\:}$, $\:R\:\approx\:11.3\hspace{0.17em}\:km)$, PSR J1614-2230 $\:(M\:=\:1.908\:\pm\:0.016\hspace{0.17em}{M}_{\odot\:}$, $\:R\:=\:9.69\:\pm\:0.2\hspace{0.17em}km)$, and the black‑widow pulsar PSR J0952-0607, which reaches $\:M\:=\:2.35\:\pm\:0.17\hspace{0.17em}{M}_{\odot\:}$ with $\:R=12.39\pm\:1.3\hspace{0.17em}\mathrm{km}.$ Gravitational‑wave events offer complementary, model‑independent constraints.

The LIGO-Virgo analysis of GW170817 suggests a radius of $\:(11.9\pm\:1.4\hspace{0.17em}\mathrm{km})$ for its two components $\:\left(M\approx\:1.27-1.45\hspace{0.17em}{M}_{\odot\:}\right)$, while later work^64^ infers $\:(R=12.9\pm\:0.8\hspace{0.17em}\mathrm{km})$ for a canonical $\:(1.4\hspace{0.17em}{M}_{\odot\:})$ neutron star. These values align with the larger radii favored by NICER and quiescent LMXBs, collectively pointing toward a moderately stiff equation of state that supports radii $\:(\gtrsim\:12\hspace{0.17em}\mathrm{km})$ at $\:(M\sim\:1.4\hspace{0.17em}{M}_{\odot\:})$ while still accommodating massive $\:\left(\gtrsim\:2.0\hspace{0.17em}{M}_{\odot\:}\right)$ configurations.

**Table 5:** Observed mass and radius of 21 pulsars.

Pulsar	References	M (M$_{ \odot }$)	*R* (km)	$\bar{\rho }$ (g cm^− 3^)
High-mass X-ray binaries				
Her X-1	^49^	0.85 ± 0.15	8.1 ± 0.41	7.564E + 014
4U 1538-52	^50^	0.87 ± 0.07	7.866 ± 0.21	8.453E + 014
LMC X-4	^51^	1.04 ± 0.09	8.301 ± 0.41	8.598E + 014
Cen X-3	^52^	1.49 ± 0.49	9.178 ± 0.13	9.114E + 014
Low-mass X-ray binaries (quiescence–thermonuclear bursts)				
EXO 1785 − 248	^53^	1.3 ± 0.2	8.849 ± 0.4	8.872E + 014
M13	^54^	1.38 ± 0.2	9.95 ± 0.28	6.625E + 014
X7	^55^	1.1 ± 0.3	12.0 ± 1.0	3.010E + 014
4U 1820-30	^56^	1.58 ± 0.3	9.11 ± 0.4	5.048E + 014
4U 1608-52	^57^	1.74 ± 0.14	9.3 ± 1.0	7.888E + 014
KS 1731 − 260	^53^	1.61 ± 0.36	10 ± 2	7.614E + 014
EXO 1745 − 248	^53^	1.4 ± 0.48	11 ± 1	6.740E + 014
4U 1724 − 207	^53^	1.81 ± 0.27	12 ± 1	4.7140E + 014
SAX J1748.9-2021	^53^	1.78 ± 0.3	7.3 ± 0.7	5.344E + 014
Millisecond pulsars				
PSR J0030 + 0451	^58^	1.44 ± 0.16	12.71 ± 1.19	3.086E + 014
PSR J0030 + 0451	^59^	1.34 ± 0.14	12.71 ± 1.19	3.085E + 014
PSR J0037-4715	^60^	1.44 ± 0.07	13.02 ± 0.27	2.707E + 014
PSR J1614-2230	^61^	1.908 ± 0.016	9.69 ± 0.2	4.107E + 014
PSR J0348 + 0432	^62^	2.01 ± 0.04	11.3	4.326E + 014
PSR J0952–0607	^63^	2.35 ± 0.17	12.39 ± 1.3	5.843E + 014
Gravitational-wave signals				
LIGO-Virgo	^64^	1.4	12.9 ± 0.8	3.084E + 014
GW170817-1	^65^	1.45 ± 0.09	11.9 ± 1.4	4.069E + 014
GW170817-2	^65^	1.27 ± 0.09	11.9 ± 1.4	3.564E + 014

**Fig. 9:**
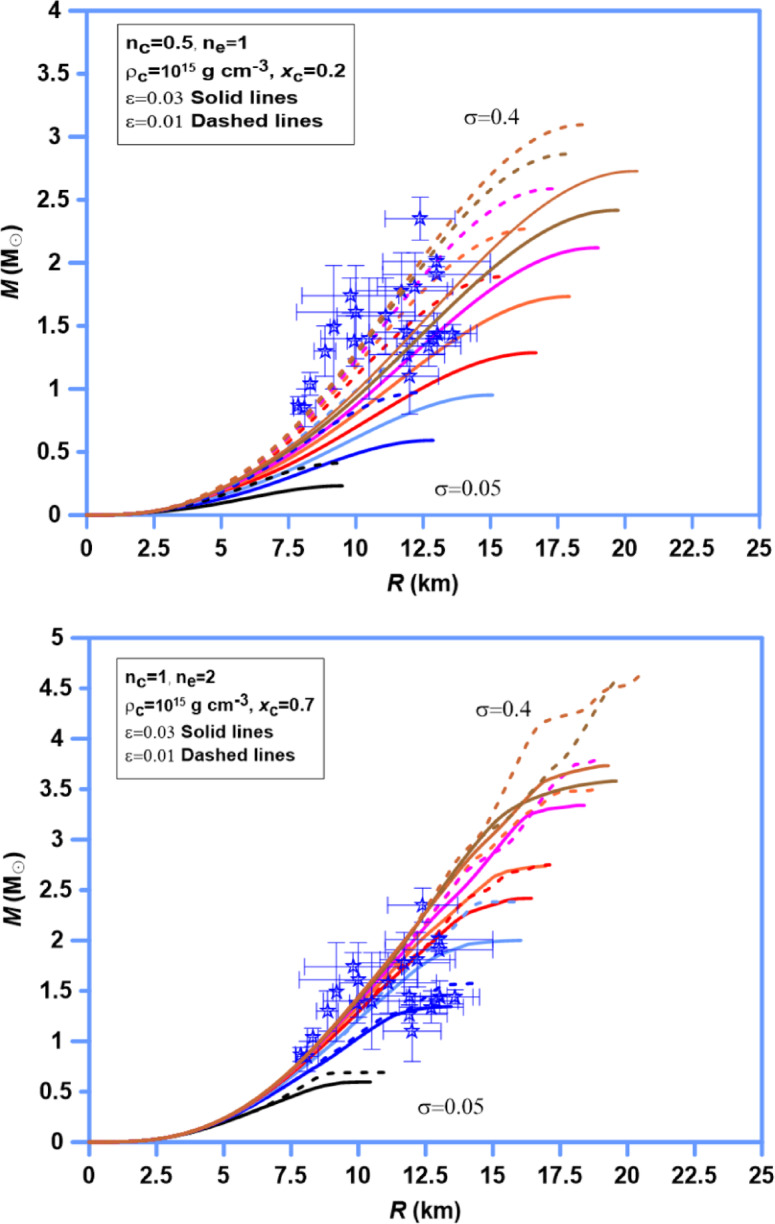
Theoretical M-R curves ($\:\sigma\:=0.05-0.4,{\rho\:}_{c}={10}^{15}g/c{m}^{3}$) vs. 21 observed NS (Table 5). Upper curve (soft: $\:{n}_{c}=0.5,\:{n}_{e}=1,\:{x}_{c}=0.2$) envelopes low-M/large-R; lower curve (stiff: $\:{n}_{c}=1,\:{n}_{e}=2,\:{x}_{c}=0.7$) fits high-M/compact; bounds data diversity.

We computed two groups of theoretical mass–radius relations for the same central density $\:({{\uprho\:}}_{c}={10}^{15}\hspace{0.17em}{\mathrm{g\:cm}}^{-3})\:$and relativistic parameter range $\:({\upsigma\:}=0.05\mathrm{-}0.5; \text{for the soft model only }\:{\upsigma\:}\:\le\:\:0.3\text{ is causal})$, but with different structural parameters. The first group of models ($\:{n}_{c}=0.5,{n}_{e}=1,{x}_{c}=0.2,\:\varepsilon=0.01\:\text{ and }\:0.03,\:\mathrm{t}\mathrm{h}\mathrm{e}\:$upper curve of Fig. 9), this model features a softer core $\:\left({n}_{c}=0.5\right)$ and a moderately stiff envelope $\:\left({n}_{e}=1\right)$, with a small core boundary $\:\left({x}_{c}=0.2\right)$. The resulting M–R relation predicts larger radii across all masses, particularly in the range $\:(1.0-2.0\hspace{0.17em}{M}_{\odot\:})$. The second group of models ($\:{n}_{c}=1,{n}_{e}=2,{x}_{c}=0.7$, $\: \varepsilon=0.01\:\text{ and }\:0.03,\:$ the lower curve of Fig. 9): This configuration has a stiffer core $\:\left({n}_{e}=1\right)$ and an even stiffer envelope $\:\left({n}_{c}=2\right)$, with a larger core $\:\left({x}_{c}=0.7\right)$. Consequently, it produces more compact stars with smaller radii for a given mass.

The observed masses and radii in Fig. 9 indicate that:

- The low‑mass, large‑radius systems (e.g., PSR J0030 + 0451, PSR J0037–4715, quiescent LMXBs) with masses $\:(\sim\:1.3-1.5\hspace{0.17em}{M}_{\odot\:})$ and radii $\:(\sim\:12-13\hspace{0.17em}\mathrm{km})$ align closely with the upper curve. This indicates that neutron stars with softer cores $\:\left({n}_{c}=0.5\right)$ and smaller core fractions ($\:{x}_{c}=0.2$) can successfully reproduce the observed larger radii at moderate masses.
- High‑mass, compact systems such as PSR J1614–2230 ($\:M\approx\:1.91\hspace{0.17em}{M}_{\odot\:}$, $\:R\approx\:9.7\hspace{0.17em}\mathrm{km}$), PSR J0348 + 0432 ($\:M\approx\:2.01\hspace{0.17em}{M}_{\odot\:}$, $\:R\approx\:11.3\hspace{0.17em}\mathrm{km}$), and PSR J0952–0607 ($\:M\approx\:2.35\hspace{0.17em}{M}_{\odot\:}$, $\:R\approx\:12.4\hspace{0.17em}\mathrm{km}$ fall closer to the lower curve. Their positions suggest that stars with stiffer equations of state ($\:{n}_{c}=1$, $\:{n}_{e}=2$) and larger core fractions $\:{x}_{c}=0.7$ are required to achieve the observed compactness at high masses.
- Intermediate systems like the thermonuclear bursters (4U 1608‑52, 4U 1820‑30) with masses ($\:1.5-1.8\hspace{0.17em}{M}_{\odot\:}$) and radii ($\:9-11\hspace{0.17em}\mathrm{km}$) lie between the two curves, indicating that their internal structure may be intermediate between the two parameter sets—perhaps with moderate core stiffness and size.

The spread of observed points between the two theoretical curves reflects the diversity of neutron‑star interiors. The upper curve ($\:{n}_{c}=0.5,{n}_{e}=1,{x}_{c}=0.2$) represents stars with a softer, more extended core and a softer envelope, naturally producing larger radii. This configuration is consistent with neutron stars that have experienced extended accretion or have a significant crustal contribution. The lower curve ($\:{n}_{c}=1,{n}_{e}=2,{x}_{c}=0.7$) corresponds to stars with a stiffer, more massive core and a stiffer envelope, leading to greater compactness. This may describe neutron stars with stronger nuclear interactions, possibly containing hyperons or other exotic components, or systems with different evolutionary histories. The fact that most observed neutron stars fall within or near the region bounded by these two curves demonstrates that the composite polytropic framework, with its tunable parameters ($\:{n}_{c},{n}_{e},{x}_{c})$, can encompass the observed M–R diversity. Specifically, in our models, we find that a stiffer core ($\:{n}_{c}$) is necessary to support the highest masses; stiffer cores ($\:{n}_{c}=1$) support higher maximum masses with smaller radii. Envelope stiffness ($\:{n}_{e}$) influences the low‑to‑intermediate mass slope: stiffer envelopes ($\:{n}_{e}=2$) yield more compact configurations across all masses. Core size ($\:{x}_{c}$) affects the mass concentration: larger cores ($\:{x}_{c}=0.7$) enhance compactness, particularly at higher masses. Relativistic parameter ($\:{\upsigma\:}$) modulates the overall compactness, with higher $\:{\upsigma\:}$ values shifting the curves downward.

## The maximum mass of neutron stars

**Fig. 10:**
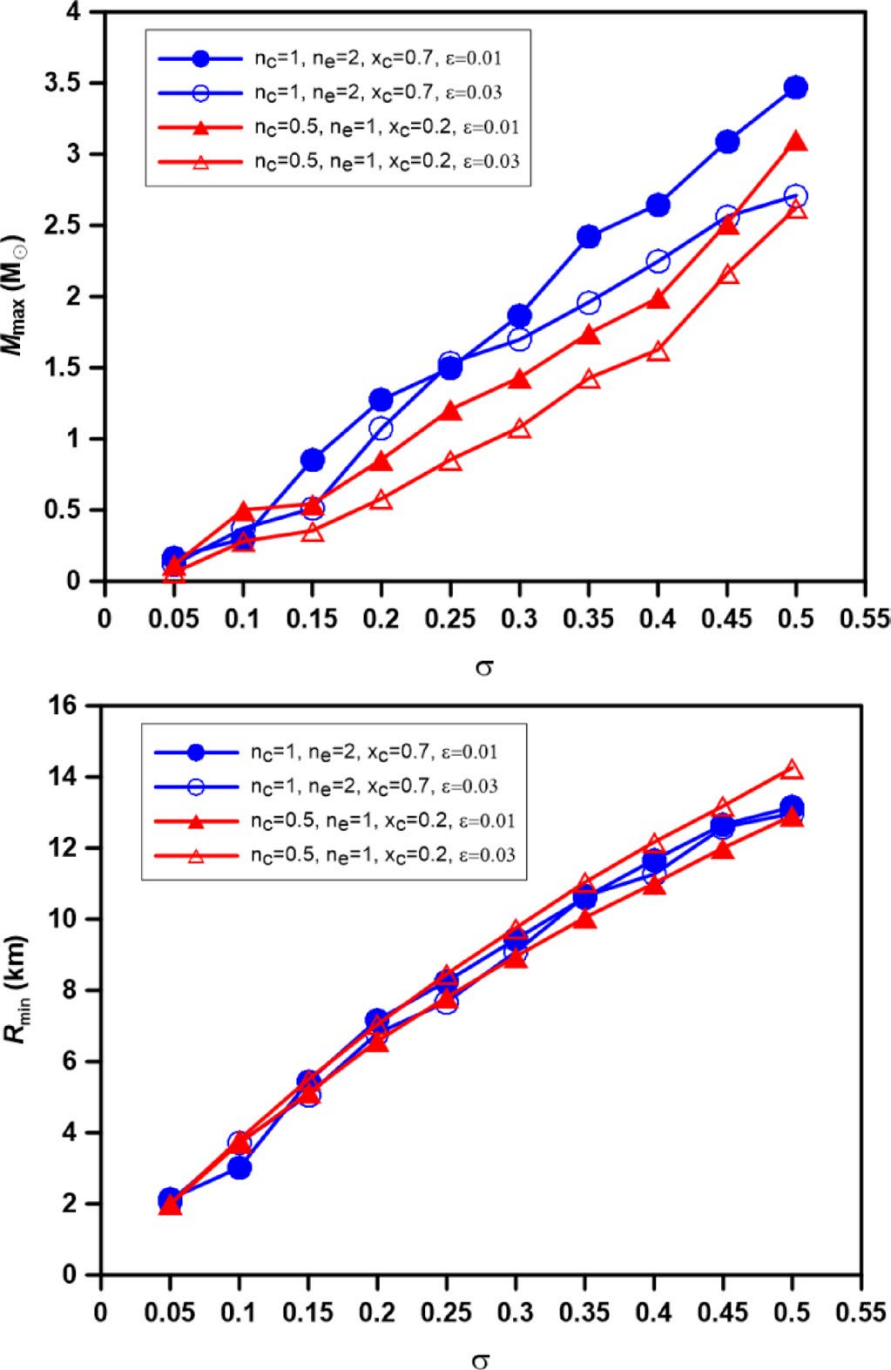
The effect of the relativistic parameter (σ) on the maximum mass; the blue curves are for the models with n_c_=1, n_e_=2, and the red curves are for n_c_=0.5, n_e_=1.

**Fig. 11:**
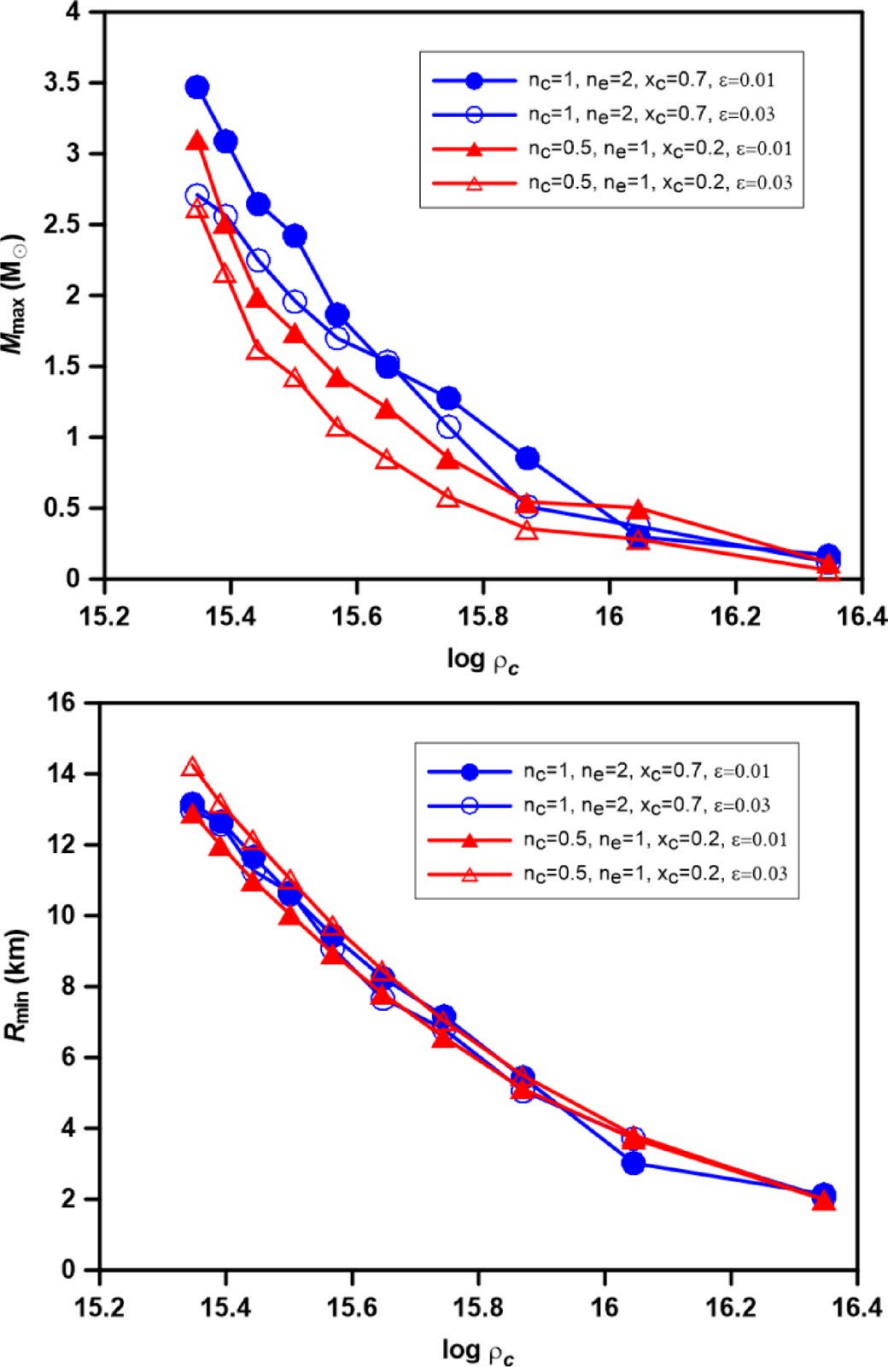
The effect of the central density (ρ_c_) on the maximum mass; the blue curves are for the models with n_c_=1, n_e_=2, and the red curves are for n_c_=0.5, n_e_=1.

**Fig. 12:**
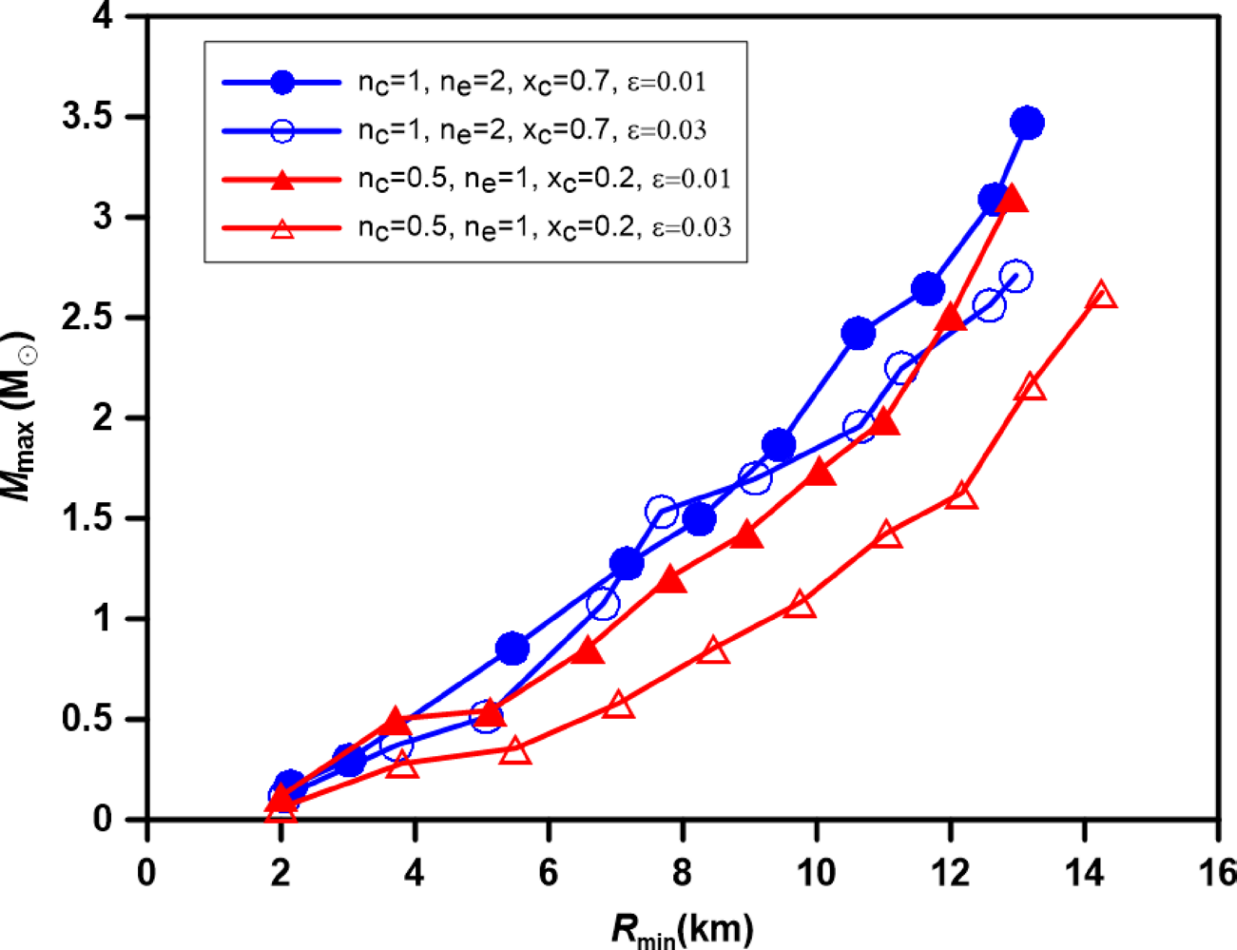
The mass-radius relation for the four models listed in Tables 6, 7, 8 and 9.

A fundamental diagnostic in the study of compact stars is the maximum gravitational mass (M_max_) that a given equation of state can support before the onset of gravitational instability, and the associated minimum radius (R_min_) of the configuration at that maximum. These two quantities serve as the most direct theoretical predictions that can be confronted with radio pulsar timing and NICER X-ray observations. We computed M_max_ and R_min_ for composite polytropic models with (n_c_ = 1, n_e_ = 2, x_c_ = 0.7) across a range of relativistic parameters, σ = 0.05–0.50, for two transition widths, ε = 0.01 and 0.03. In addition, we computed two further groups of models with a softer core configuration (n_c_ = 0.5, n_e_ = 1, x_c_ = 0.2). Tables 6 and 7 list the computed structural parameters for the stiffer-core models (n_c_ = 1, n_e_ = 2), while Tables 8 and 9 present the corresponding results for the softer-core models (n_c_ = 0.5, n_e_ = 1). Figure 10 shows the effect of the relativistic parameter on the maximum mass for the two transition widths, 0.01 and 0.03. Figure 11 shows how the maximum mass changes with central density for the same two models. Figure 12 presents the mass–radius relation, comparing the two transition widths.

**Table 6:** Maximum mass of the composite polytropic model with n_c_ = 1, n_e_ = 2, ε = 0.01, x_c_ = 0.7, P_c_=10^36^ dyne cm^− 2^ (Model 3).

σ	ρ_c_	x_s_	ν (x_s_)	M_max_ (M$_{ \odot }$)	*R*_min_ (km)
0.05	2.22 × 10^16^	2.7803	1.9647 ± 0.2314	$\:0.1683$	$\:2.1437$
0.1	1.11 × 10^16^	2.5361	1.4051 ± 0.2059	$\:0.2989$	$\:3.0168$
0.15	7.40 × 10^15^	2.3352	1.3550 ± 0.0483	$\:0.8533$	$\:5.4464$
0.2	5.55 × 10^15^	2.2131	1.0097 ± 0.1258	$\:1.2755$	$\:7.1717$
0.25	4.44 × 10^15^	2.1254	0.8224 ± 0.1316	$\:1.4986$	$\:8.2550$
0.3	3.70 × 10^15^	2.0161	0.7280 ± 0.1115	$\:1.8698$	$\:9.4433$
0.35	3.17 × 10^15^	1.8789	0.6173 ± 0.0745	$\:2.4210$	$\:10.6120$
0.4	2.77 × 10^15^	1.8182	0.5355 ± 0.0653	$\:2.6480$	$\:11.6711$
0.45	2.46 × 10^15^	1.7833	0.4623 ± 0.0693	$\:3.0913$	$\:12.6589$
0.5	2.22 × 10^15^	1.7675	0.4509 ± 0.0655	$\:3.4734$	$\:13.1525$

**Table 7:** Maximum mass of the composite polytropic model with n_c_ = 1, n_e_ = 2, ε = 0.03, x_c_ = 0.7, P_c_=10^36^ dyne cm^− 2^ (Model 4).

σ	ρ_c_	x_s_	ν (x_s_)	M_max_ (M$_{ \odot }$)	*R*_min_ (km)
0.05	2.22 × 10^16^	2.8229	2.1224 ± 0.3596	0.1197	2.0598
0.1	1.11 × 10^16^	2.5635	1.5945 ± 0.2301	0.3713	3.7267
0.15	7.40 × 10^15^	2.3231	1.0865 ± 0.1947	0.5111	5.0616
0.2	5.55 × 10^15^	2.2024	0.9611 ± 0.1648	1.0731	6.8020
0.25	4.44 × 10^15^	2.1145	0.8469 ± 0.1406	1.5351	7.6756
0.3	3.70 × 10^15^	2.0389	0.7609 ± 0.0924	1.6977	9.0842
0.35	3.17 × 10^15^	1.9502	0.5963 ± 0.1151	1.9606	10.650
0.4	2.77 × 10^15^	1.8545	0.5678 ± 0.0693	2.2472	11.264
0.45	2.46 × 10^15^	1.8185	0.4905 ± 0.0714	2.5622	12.581
0.5	2.22 × 10^15^	1.7495	0.4627 ± 0.0488	2.7104	12.975

**Table 8:** Maximum mass of the composite polytropic model with n_c_ = 0.5, n_e_ = 1, ε = 0.01, x_c_ = 0.2, P_c_=10^36^ dyne cm^− 2^ (Model 5).

σ	ρ_c_	x_s_	ν (x_s_)	M_max_ (M$_{ \odot }$)	*R*_min_ (km)
0.05	2.22 × 10^16^	2.8684	1.8442 ± 0.0208	$\:0.1170$	$\:1.9920$
0.1	1.11 × 10^16^	2.6692	1.4824 ± 0.0241	$\:0.5019$	$\:3.7073$
0.15	7.40 × 10^15^	2.5073	1.2652 ± 0.0213	$\:0.5439$	$\:5.1219$
0.2	5.55 × 10^15^	2.3680	1.0531 ± 0.0213	0.8553	6.57748
0.25	4.44 × 10^15^	2.2486	0.9149 ± 0.0044	$\:1.2077$	$\:7.8080$
0.3	3.70 × 10^15^	2.1491	0.7848 ± 0.0157	$\:1.4316$	$\:8.9542$
0.35	3.17 × 10^15^	2.0654	0.6858 ± 0.0105	$\:1.7422$	$\:10.0470$
0.4	2.77 × 10^15^	1.9899	0.6098 ± 0.0101	$\:1.9900$	$\:11.0070$
0.45	2.46 × 10^15^	1.9205	0.5433 ± 0.0111	$\:2.5144$	$\:12.0034$
0.5	2.22 × 10^15^	1.8593	0.4893 ± 0.0103	$\:3.1065$	$\:12.9119$

**Table 9:** Maximum mass of the composite polytropic model with n_c_ = 0.5, n_e_ = 1, ε = 0.03, x_c_ = 0.2, P_c_=10^36^ dyne cm^− 2^ (Model 6).

σ	ρ_c_	x_s_	ν (x_s_)	M_max_ (M$_{ \odot }$)	*R*_min_ (km)
0.05	2.22 × 1016	2.8796	0.9632 ± 0.0950	$\:0.0611$	$\:1.9997$
0.1	1.11 × 1016	2.7438	0.8286 ± 0.0820	$\:0.2805$	$\:3.8108$
0.15	7.40 × 1015	2.6406	0.7790 ± 0.0770	$\:0.3553$	$\:5.5011$
0.2	5.55 × 1015	2.5369	0.7148 ± 0.0506	$\:0.5805$	$\:7.0464$
0.25	4.44 × 1015	2.4378	0.6474 ± 0.0642	$\:0.8546$	$\:8.4647$
0.3	3.70 × 1015	2.34153	0.5929 ± 0.0654	$\:1.0817$	$\:9.7555$
0.35	3.17 × 1015	2.26937	0.5619 ± 0.0489	$\:1.4274$	$\:11.0392$
0.4	2.77 × 1015	2.19009	0.4984 ± 0.0471	$\:1.6264$	$\:12.1661$
0.45	2.46 × 1015	2.10911	0.4674 ± 0.0515	$\:2.1629$	$\:13.1817$
0.5	2.22 × 1015	2.05293	0.4135 ± 0.0396	$\:2.6250$	$\:14.2565$

The most striking feature of Tables 6 and 7 (n_c_ = 1, n_e_ = 2) is the strong monotonic increase in M_max_ with increasing σ. For the sharp-transition model (ε = 0.01), M_max_ rises from 0.168 M$_{ \odot }$ at σ = 0.05 to 3.47 M$_{ \odot }$ at σ = 0.50, a factor of approximately 21. The smooth-transition model (ε = 0.03) shows a similar trend, with M_max_ increasing from 0.12 M$_{ \odot }$ to 2.71 M$_{ \odot }$ over the same σ range. This behaviour is physically expected and consistent with the general-relativistic structure of the TOV equation: as σ increases, the ratio of central pressure to rest-mass energy density grows, and the star’s self-gravity is balanced by stiffer pressure support, allowing more massive equilibrium configurations. Importantly, σ also governs the density at which these maximum masses are achieved. As σ increases from 0.05 to 0.50, the central density at the maximum-mass configuration decreases from ~ 2.2 × 10^16^ g cm − 3 to ~ 2.2 × 10^15^ g cm^− 3^, a decrease of roughly one order of magnitude. This inverse relationship between σ and ρ_c_ at maximum mass reflects the well-known result that stiffer equations of state (larger σ at a given density) reach their maximum mass at lower central densities, a consequence of general relativistic pressure feedback in the TOV equation. These monotonic trends in M_max_ and ρ_c_ with σ are illustrated in Figs. 10 and 11, respectively.

A comparison of the two models at identical σ reveals that the sharp transition (ε = 0.01) consistently yields higher maximum masses and larger minimum radii than the smooth transition (ε = 0.03). At σ = 0.40, for example, the sharp model gives M_max_ = 2.648 M$_{ \odot }$ with R_min_ = 11.67 km, compared to 2.47 M$_{ \odot }$ and 11.26 km for the smooth model, a difference of $\sim$18% in mass. This systematic difference arises from the nature of the compositional transition: a sharper core–envelope boundary (smaller ε) concentrates the mass more efficiently within the relativistic core, where the stiffer EoS (n_c_ = 1) is active over a larger fraction of the stellar volume. The extended dominance of the stiffer core index effectively hardens the global equation of state, pushing the maximum mass upward. Conversely, the smoother transition (ε = 0.03) distributes the compositional change over a broader density range, diluting the contribution of the stiffer core and resulting in a softer effective EoS with correspondingly lower maximum masses. This finding echoes theoretical predictions for sharp hadron–quark phase transitions, in which an abrupt EoS change at the core boundary typically yields more massive configurations than a smooth crossover with equivalent endpoint properties. The systematic offset between the two models across the full σ range is shown directly in Fig. 10, where the blue curve with ε = 0.01 lies consistently above the blue curve with ε = 0.03 at every relativistic parameter value.

The observational lower bound on the maximum mass of a neutron star is set by the most massive pulsars with reliably measured masses. The discovery of PSR J1614 − 2230 with M = 1.908 ± 0.016 M$_{ \odot }$, ^61^ provided the first definitive demonstration that the maximum mass exceeds 1.9 M$_{ \odot }$, ruling out a wide class of soft equations of state, including those with pure hyperonic cores. PSR J0348 + 0432 subsequently strengthened this constraint at M = 2.01 ± 0.04 M$_{ \odot }$^62^, PSR J0740 + 6620 at M = 2.08 ± 0.07 M$_{ \odot }$^67,68^, and most recently PSR J0952 − 0607 at M = 2.35 ± 0.17 M$_{ \odot }$^63^, which currently sets the highest reliably measured neutron star mass. Our models achieve M_max_ ≥ 2.0 M$_{ \odot }$ at σ ≥ 0.35 for ε = 0.01 and at σ ≥ 0.40 for ε = 0.03. The sharp-transition model with σ = 0.40–0.45 yields M_max_ ≈ 2.65–3.09 M$_{ \odot }$, comfortably encompassing the masses of PSR J0740 + 6620 and PSR J0952 − 0607. The smooth-transition model at σ = 0.45–0.50 yields M_max_ ≈ 2.56–2.71 M$_{ \odot }$, consistent with the PSR J0952 − 0607 measurement within uncertainties. Crucially, our models also satisfy the causality condition throughout the stellar interior for the stiffer-envelope configuration (n_c_ = 1, n_e_ = 2), so these high-mass configurations are physically admissible and not artefacts of superluminal sound speeds.

As demonstrated from Fig. 12, the values of R_min_ at the maximum mass configurations range from 10.6 to 13.2 km for ε = 0.01 and 10.7–13.0 km for ε = 0.03, consistent with the radius range 11.9–13.0 km favoured by NICER measurements and the GW170817 tidal deformability constraint^65^, which disfavours radii below ~ 10.5 km for a 1.4 M$_{ \odot }$ star. The classical result of^69^ for a free degenerate neutron gas yields M_max_ ≈ 0.71 M$_{ \odot }$, a value far below the observed neutron star masses and long understood to reflect the softness of the non-interacting neutron EoS. The single-polytrope relativistic results of^7^ for *n* = 1 yield M_max_ ≈ 0.56–0.82 M$_{ \odot }$, depending on σ, which is significantly below our composite model predictions even at similar σ values. This demonstrates the physical advantage of the composite formulation: by assigning a stiffer index to the core (Γ_c_ = 2 for n_c_ = 1) and a softer index to the envelope (Γ_e_ = 1.5 for n_e_ = 2), the composite model simultaneously achieves higher central pressure support and a realistic envelope structure, producing maximum masses substantially in excess of any uniform-polytrope prediction. Rhoades and Ruffini^70^ derived the causal upper bound M_max_ ≤ 3.2 M$_{ \odot }$ from the twin requirements of general relativity and subluminal sound speeds, later refined to $\sim$2.9 M$_{ \odot }$ by^71^.

Our sharp-transition model reaches M_max_ = 3.47 M$_{ \odot }$ at σ = 0.50, which approaches the Rhoades–Ruffini limit. This should be interpreted with caution: at σ = 0.50, the central density ($\sim$2.2 × 10^15^ g cm^− 3^, $\sim$10ρ₀) significantly exceeds the density range over which the purely hadronic polytropic EoS is physically justified, and the sound speed approaches its causal limit. However, values in the range σ = 0.35–0.45, which yield M_max_ ≈ 2.4–3.1 M$_{ \odot }$, correspond to central densities ρ_c_ $\sim$2.5–3.2 × 10^15^ g cm^− 3^ ($\sim$ 5–7ρ₀), which fall within the regime accessible to current nuclear EoS calculations, particularly those employing density-dependent relativistic mean-field models. Two representative examples are DD2 and GM1. DD2 (Density-Dependent parametrisation 2^72^) is a relativistic mean-field EoS in which the meson–nucleon coupling constants are functions of the local baryon density, calibrated simultaneously to finite-nucleus binding energies, charge radii, and neutron skin thicknesses across a wide range of nuclei. At nuclear saturation density ρ₀ = 0.149 fm^-3^ it yields an incompressibility K₀ = 243 MeV, a symmetry energy J = 31.7 MeV, and an effective nucleon mass m*/m_n_ = 0.56, predicting a maximum neutron star mass M_max_ ≈ 2.42 M$_{ \odot }$ and a canonical radius R₁.₄ ≈ 13.2 km. The density-dependent couplings naturally capture the in-medium modification of the nucleon–nucleon interaction at super-nuclear densities, making DD2 one of the most widely used EoS in core-collapse supernova and binary neutron star merger simulations. GM1 (Glendenning–Moszkowski parametrisation 1^73^) is a standard RMF EoS with fixed (density-independent) meson–nucleon coupling constants calibrated to four nuclear saturation properties: binding energy E/A − m_n_c² = −16.3 MeV, incompressibility K₀ = 300 MeV, symmetry energy J = 32.5 MeV, and effective nucleon mass m*/m_n_ = 0.70. The higher incompressibility makes GM1 a stiffer EoS than DD2, predicting M_max_≈ 2.37 M$_{ \odot }$ and R₁.₄ ≈ 13.8 km; it has been extensively used in studies of hyperonic neutron stars, where the appearance of strange baryons above $\sim$2ρ₀ softens the EoS and lowers the maximum mass. Both DD2 and GM1 correspond to polytropic core indices n_c_ ≈ 0.87–0.91 and adiabatic indices Γ_c_ ≈ 2.1–2.15, consistent with the n_c_ established by fitting nuclear EoS.

Turning to the softer-core models (n_c_ = 0.5, n_e_ = 1, x_c_ = 0.2), Tables 8 and 9 reveal a distinct structural behaviour compared with the stiffer-core configuration. For the sharp-transition model (n_c_ = 0.5, n_e_ = 1, ε = 0.01), M_max_ increases from 0.117 M$_{ \odot }$ at σ = 0.05 to 3.107 M$_{ \odot }$ at σ = 0.50, a factor of approximately 27. The corresponding smooth-transition model (ε = 0.03) yields M_max_ rising from 0.061 M$_{ \odot }$ to 2.625 M$_{ \odot }$ over the same σ range. Both softer-core models, therefore, span a comparable mass range to the stiffer-core counterparts but with systematically larger minimum radii: R_min_ reaches 14.26 km at σ = 0.50 for ε = 0.03, approximately 8 per cent larger than the corresponding n_c_ = 1 model (12.98 km). This is physically expected: the softer core (n_c_ = 0.5 corresponds to Γ_c_ = 3, a stiff pressure–density relation, but the small core fraction x_c_ = 0.2 means this stiff region occupies only the innermost 20 per cent of the star in dimensionless coordinates) produces a more extended stellar configuration with a larger envelope fraction, driving the radius upward relative to the large-core models.

Comparatively, at σ = 0.40 the stiffer-core sharp-transition model (n_c_ = 1, ε = 0.01) gives M_max_ = 2.648 M$_{ \odot }$ with R_min_ = 11.67 km, while the softer-core sharp-transition model (n_c_ = 0.5, ε = 0.01) gives M_max_ = 1.982 M$_{ \odot }$ with R_min_ = 11.05 km. The stiffer-core model thus supports a 34 per cent higher maximum mass at identical σ, confirming that the core size fraction x_c_ and the envelope stiffness index n_e_ together determine the global mass limit more decisively than the core stiffness index n_c_ alone. The softer-core models reach the observationally critical threshold of M_max_ ≥ 2.0 M$_{ \odot }$ at σ ≥ 0.45 for both ε values, compared with σ ≥ 0.35 (ε = 0.01) and σ ≥ 0.40 (ε = 0.03) for the stiffer-core models.

An important caveat applies specifically to the n_c_ = 0.5, n_e_ = 1 models. As noted in Stability analysis, the causality condition v_s_ < c is violated for this configuration at σ > 0.3, because the polytropic index n_c_ = 0.5 (Γ_c_ = 3) produces a pressure–density slope that drives the sound speed above c at super-nuclear densities corresponding to σ  ${ \gtrsim }$.3. Consequently, only the models with σ ≤ 0.3 in Tables 8 and 9 are strictly causal and physically admissible; those with σ = 0.35–0.50 are presented for mathematical completeness and to illustrate the structural trends but must be regarded as exceeding the physical applicability of a purely hadronic polytropic description. Within the causal range $\left( {\sigma \le 3} \right)$, the softer-core models predict M_max_ ≈ 0.54–1.43 M$_{ \odot }$ (ε = 0.01) and M_max_ ≈ 0.36–1.08 M$_{ \odot }$ (ε = 0.03), with corresponding minimum radii of 5.1–8.95 km and 5.5–9.76 km, respectively. These values are below the observational threshold 2 M$_{ \odot }$. Still, they are physically consistent with lower-mass neutron stars and massive white dwarfs, and they confirm that the composite polytrope with a soft core and small core fraction naturally describes less compact stellar configurations than the stiffer-core, large-core-fraction models. Table 10 places the present results for all four model groups in the context of key theoretical and observational benchmarks from the literature.

**Table 10:** Maximum mass comparison with theory and observation.

Model / source	M_max_ (M$_{ \odot }$)	R_m_in (km)	References
Free neutron gas (OV)	0.71	—	^69^
General upper bound (GR + causality)	3.2	—	^70^
Revised upper bound	2.9	—	^71^
PSR J1614 − 2230 (Shapiro delay)	1.97 ± 0.04	—	^66^
PSR J0348 + 0432	2.01 ± 0.04	—	^62^
PSR J0740 + 6620 (NICER)	2.08 ± 0.07	—	^67^
PSR J0952 − 0607 (Black widow)	2.35 ± 0.17	12.4 ± 1.3	^63^
PSR J0740 + 6620 (NICER + XMM)	2.08	12.35 ± 0.75	^68^
Relativistic polytrope *n* = 1	0.56–0.82	—	^7^
Model 3, σ = 0.35–0.50	2.42–3.47	10.6–13.2	This study
Model 4, σ = 0.35–0.50	1.96–2.71	10.7–13.0	This study
Model 5, causal σ ≤ 0.3.	0.54–1.43	5.1–8.95	This study
Model 6, causal σ ≤ 0.3.	0.36–1.08	5.5–9.76	This study

OV = Oppenheimer–Volkoff. NICER = Neutron Star Interior Composition Explorer. ρ₀ = 2.3 × 10¹⁴ g cm^-3^.

Although polytropic models are parametric approximations rather than first-principles nuclear calculations, they have a well-established and quantifiable relationship to realistic nuclear equations of state, and it is important to make this connection explicit for the composite model introduced here. For a single-component polytrope, the adiabatic index Γ = 1 + 1/n directly governs the pressure–density slope and is related to the nuclear incompressibility modulus K₀ at saturation density ρ₀ by K₀ = 9ρ₀ dP/dρ|ρ₀, so that a stiffer EoS with higher K₀ corresponds to a lower polytropic index n and a higher Γ. In the composite framework adopted here, the core index n_c_ maps onto the high-density behaviour of the nuclear EoS above saturation. In contrast, the envelope index n_e_ encodes the low-to-intermediate density behaviour near and below ρ₀.

Quantitatively, for the soft nuclear EoS SLy4^74^, a piecewise polytropic fit in the density range 10¹⁴–10¹⁵ g cm^-3^ yields Γ_c_ ≈ 3.0 (n_c_ ≈ 0.5), consistent with our Models 5 and 6, while the intermediate-stiffness EoS APR^75^ yields Γ_c_ ≈ 2.4 (n_c_ ≈ 0.7) and the stiffer nuclear EoS NL3^76^ yields Γ_c_ ≈ 2.0 (n_c_ ≈ 1.0), consistent with our Models 3 and 4. At sub-saturation densities corresponding to the stellar envelope, all three EoS converge toward Γ_e_ ≈ 1.5–2.0 (n_e_ ≈ 1.0–2.0), precisely the range spanned by our envelope index.

The polytropic constant K is similarly calibrated: requiring $P\left( {\rho _{0} } \right)\, = \,K\rho _{0} ^{{{{1 + 1} \mathord{\left/ {\vphantom {{1 + 1} {nc}}} \right. \kern-\nulldelimiterspace} {nc}}}}$ to match the pressure predicted by a given EoS at nuclear saturation density ρ₀ = 2.3 × 10¹⁴ g cm^-3^ uniquely determines K for each (n_c_, EoS) pair, removing it as a free parameter and anchoring the model to nuclear data. Table 11 below summarises this calibration for three representative EoS spanning the soft-to-stiff range currently consistent with NICER and gravitational-wave constraints; the resulting polytropic parameters fall entirely within the range explored in our Monte Carlo study, confirming that the parameter space we probe is not arbitrary but encompasses the full range of physically motivated nuclear EoS currently considered viable. The parameters in Table 11 clearly link the abstract CTOV model to concrete, empirically proven nuclear equations of state.

**Table 11:** Piecewise polytropic parameters (n_c_, n_e_, K) obtained by fitting three nuclear EoS in the density range 10¹⁴–10¹⁵ g cm^-3^, following the method of^77^.

EoS	Type	Γc	nc	Γe	ne	K (CGS)	M_max_ (M$_{ \odot }$)	*R*_min_ (km)
SLy4	Soft hadronic	3.01	0.50	1.79	1.26	5.2 × 10⁹	2.05	11.7
APR	Moderate hadronic	2.43	0.70	1.65	1.54	4.8 × 10⁹	2.20	11.9
NL3	Stiff hadronic	2.00	1.00	1.48	2.08	7.3 × 10⁹	2.79	14.9

M_max_ and R_min_ are the maximum mass and radius at 1.4 M$_{ \odot }$ predicted by each EoS for reference. All three EoS maps onto the parameter space explored in our Monte Carlo study.

## Conclusions

We have developed a stochastic numerical framework for solving the relativistic composite polytropic equations that describe stratified compact stars. By introducing a smoothly varying polytropic index $\:n\left(r\right)$ within the Tolman–Oppenheimer–Volkoff formalism, we constructed composite models that better represent the layered interiors of neutron stars and white dwarfs. The resulting CTOV equations are integrated using a Monte Carlo scheme, yielding ensemble solutions with inherent uncertainty quantification and enabling probabilistic exploration of parameter space.

The main findings of this study are as follows:

1. The Monte Carlo method was validated by comparison with Runge–Kutta methods. The MC estimates of the first zero of the Emden function ($\:{x}_{s}$) agree with RK reference solutions within 0.025–0.066 for relativistic parameters $\:\sigma\:$ = 0–0.5. The ensemble-averaged profiles of the Emden function $\:\theta\:\left(x\right)$ and mass function $\:\nu\:(x$) showed minimal scatter in the core and smooth convergence toward the stellar surface.
2. The transition width ($\:\varepsilon\:$) plays a major structural role. Sharper transitions ($\:\varepsilon\:\:=\:0.01$) produce more compact stars than smoother transitions ($\:\varepsilon\:\:=\:0.03$). For example, Model 1 ($\:{n}_{c}=0.5,{n}_{e}=1,{x}_{c}=0.2$) yields ($\:{{v}_{s}(x}_{s})/{x}_{s}$ = 0.756) compared with ($\:{{v}_{s}(x}_{s})/{x}_{s}$ = 0.38) at ($\:\sigma\:$ = 0).
3. Models with stiffer and larger cores ($\:{n}_{c}=1,{n}_{e}=2,{x}_{c}=0.7$) produce smaller radii and higher compactness, successfully reproducing high-mass pulsars such as PSR J1614–2230.
4. All computed models satisfy essential physical requirements, including positivity and monotonic decrease of density and pressure, dynamical stability ($\:{\Gamma\:}>\frac{4}{3}$), causality ($\:{v}_{s}<c$), and the standard energy conditions.
5. The derived mass–radius relations reproduce the observed diversity of neutron stars. Softer composite models align with large-radius objects such as PSR J0030 + 0451 ($\:R\:\sim\:\:12.7$ km). Stiffer models match compact high-mass pulsars such as PSR J1614–2230 ($\:R\:\sim\:\:9.7$ km). The theoretical curves bound most observed neutron-star data, indicating that variations in core stiffness, envelope structure, and transition sharpness can explain the observed M–R spread.
6. A major result is the maximum mass prediction. For stiff composite configurations ($\:{n}_{c}=1,{n}_{e}=2,{x}_{c}=0.7$), the maximum mass increases strongly with σ. The sharp-transition model ($\:\varepsilon\:\:=\:0.01$) reaches $\:{M}_{max}\:\approx\:\:3.47,{M}_{\odot\:}\:$at $\:\sigma\:=\:0.50$, approaching the causal upper bound. The smooth-transition model ($\:\varepsilon\:\:=\:0.03$) yields $\:{M}_{max}\approx\:2.71\:{M}_{\odot\:}$with minimum radii $\:{R}_{min}\approx\:10.6-13.2\:\text{ km}$, consistent with NICER and gravitational-wave constraints. These models satisfy the observational requirement of neutron stars above $\:2{M}_{\odot\:}$, reproducing systems such as PSR J0348 + 0432 and PSR J0952 − 0607.
7. Unlike deterministic solvers, the MC approach provides both a mean solution and a statistical ensemble, enabling direct estimation of numerical uncertainties and analysis of degeneracies in the mass–radius relation. By treating ($\:{\rho\:}_{c}$), ($\:{x}_{c}$), ($\:\varepsilon\:$), and ($\:\sigma\:$) as random variables, the framework naturally supports probabilistic inference with astrophysical priors and yields posterior distributions for key structural diagnostics.
8. This work serves as a methodological proof of concept for applying stochastic numerical solvers to relativistic stellar structure, extending MC integration from the Lane–Emden system to the more complex CTOV equations. The model remains limited by its use of simplified polytropic approximations and assumptions such as isotropic pressure, and it does not include detailed nuclear physics (e.g., hyperons, superfluidity, or sharp phase transitions). Additionally, the reported uncertainties reflect numerical sampling effects rather than full physical parameter uncertainties. Future work will focus on incorporating realistic nuclear equations of state, extending the framework to MCMC-based parameter estimation constrained by NICER data, and developing a more comprehensive statistical tool for constraining the neutron-star equation of state using current and future observations.

## Data Availability

The datasets used and/or analysed during the current study are available from the corresponding author on reasonable request.

## Appendix A: general theory of monte carlo integration

This appendix provides the general mathematical background for the Monte Carlo (MC) integration method used in Sect.  3. The material is standard and included here for completeness.

### Fundamentals of MC integration

For a definite integral of a function $\:\:f\left(t\right)\:$over the interval [a, b], we have^39–42^.

34 $$I = \int_{a}^{b} f \left( t \right)dt,$$

the mean-value theorem provides the approximation $\:I\approx\:\left(b-a\right)$ $\left\langle f \right\rangle$, where $\left\langle f \right\rangle$ is the average value of $\:f$ over the interval. The MC method estimates this average by sampling $\:N$ random points t_i_, uniformly distributed in [a, b]:

35 $$\left\langle f \right\rangle \approx \:\frac{1}{N}\sum\limits_{{i = 1}}^{N} {f\left( {t_{i} } \right).}$$

Thus, the integral is approximated as:

36 $$I \approx \:\frac{{\left( {b - a} \right)}}{N}\sum \: _{{i = 1}}^{N} f\left( {t_{i} } \right).$$

A more robust variant, the hit-or-miss method, is employed when $\:f\left(t\right)$ is bounded. If $\:M$ and $\:R$ are the upper and lower bounds of $\:f\left(t\right)$ on [a, b] (i.e., $\:R\le\:f\left(t\right)\le\:M$), random points ($\:{t}_{i},{y}_{i}$) are generated uniformly in the rectangle ($\:\left[a,b\right]\times\:\left[R,M\right]$). The integral is then proportional to the fraction of points falling under the curve $\:f\left(t\right)$:

37 $$\int_{a}^{b} f \left( t \right)dt = M\left( {b - a} \right)\frac{S}{N}.$$

Where *S* is the count of hits. This geometric approach forms the basis for extending MC to differential equations.

### Adaptation to ordinary differential equations (ODEs)

The MC integration framework can be adapted to solve initial value problems of the form:

38 $$y^{{\prime \:}} \left( t \right) = F\left( {t,y} \right),y\left( {t_{0} } \right) = y_{0} ,$$

by treating the derivative as an integrand. The solution at a point $\:t$ is given by:

39 $$y\left( t \right) = y\left( {t_{0} } \right) + \int_{{t_{0} }}^{t} F \left( {\tau ,y\left( {\tau \:} \right)} \right)d\tau .$$

The MC algorithm proceeds iteratively over a discretised domain, $\:{t}_{0},\:{t}_{1},\:\dots\:,\:{t}_{m}$, with a step size $\:dt$. At each step from $\:{t}_{i}$ to $\:{t}_{i+1}$, the integral of $\:F$ is approximated using the hit-or-miss method:

1. Determine numerical bounds $\:{M}_{1`}$ (upper) and $\:{R}_{1}$ (lower) for $\:F(t,\:y)$ over the current step.
2. Generate random samples uniformly in $\:[0,{\:M}_{1}]$ or $\:[{R}_{1},\:0]$ depending on the sign of $\:F$.
3. Count the number $\:{S}_{1}$ of samples that satisfy the hit condition.
4. Update the solution:

- If F ≥ 0 : y_i+1_ = y_i_ + M_1_· (S₁/N) · Δt.
- If F ≤ 0 : y_i+1_ = y_i_ + R_1_ · (S₁/N) · Δt.

The same procedure is applied recursively if the ODE system is of higher order, by first reducing it to a system of first-order equations. The error of the MC estimate scales as $\:O(1/\sqrt{N}$), independent of the dimension of the integral, which makes the method particularly attractive for high-dimensional problems.

## Appendix B: computational sequence of the MC solver for the CTOV equations

This appendix provides the detailed implementation steps of the Monte Carlo solver for the CTOV equations, as summarised in Sect.  3.

### Initialization and parameter setup

1. Define physical constants and parameters: Set the relativistic parameter σ, central density $\:{\rho}_{c}$, core polytropic index $\:{n}_{c}$, and envelope polytropic index $\:{n}_{e}$, and core size parameter $\:{x}_{c}$.
2. Composite Logic: To enable a variable polytropic index $\:n\left(x\right)$ controlled by a hyperbolic tangent function (controlled by ε and $\:x/{x}_{\mathrm{s}}$), compute the transition parameters $\:{{\upeta\:}}_{0}=\frac{{n}_{c}+{n}_{e}}{2}$ and $\:{{\upeta\:}}_{1}={n}_{c}-{{\upeta\:}}_{0}$.
3. Determine MC bounds: Estimate the search space for the MC sampler:

- For θ (dimensionless density): bounds $\:\left[{R}_{\theta\:},{M}_{\theta\:}\right]$, where $\:{R}_{\theta\:}$< 0 and $\:{M}_{\theta\:}$> 0.
- For ν (dimensionless mass): bounds $\:\left[0,{M}_{{\upnu\:}}\right]$, where $\:{M}_{{\upnu\:}}$> 0.

### B.2 External loop: parallel simulations

To explore the parameter space and the surface boundary $\:{x}_{\mathrm{s}}$, the solver performs $\:m$ independent runs:

- Parallelisation: Use a parallel computing environment (e.g., do Parallel in R) to utilise multiple CPU cores.
- Sampling $\:{x}_{\mathrm{s}}$: For each run, select 100 candidate surface positions $\:{x}_{\mathrm{s}}$, from a uniform distribution.
- Pre-generate random samples: Create uniform random numbers for the sample arrays using $\:N={2\times\:10}^{6}$ within the MC bounds for θ and ν.

### B.3 Inner loop: MC integration solver

For each candidate $\:{x}_{\mathrm{s}}$, the system is integrated from the center $\:x={10}^{-6}\:$to the surface $\:x={x}_{\mathrm{s}}$ $\::$.

**A**. Core boundary conditions:

Set the starting values to $\:x={10}^{-6}$, $\:\theta\:=1.0$ (normalised central density), and $\:\nu\:=0$ (zero mass at the center).

**B.** Stochastic gradient sampling at each step $\:k$:

1. Evaluate the CTOV equations to compute the theoretical derivatives $\:d\theta\:$ and $\:d\nu\:.$.
2. MC Sampling for $\:\theta\:$:

- If $\:d\theta\:\ge\:0$: Count the number of pre-generated samples in the positive sample array of $\:\theta\:$ are $\:\le\:d\theta\:$.
- If $\:d\theta\:\le\:0$: Count the number of pre-generated samples in the negative sample array of $\:\theta\:$ are $\:\ge\:d\theta\:$.
- Update: $$\:{\theta\:}_{k}\:=\:{\theta\:}_{k-1}\:+\:Bound\times\:\frac{Count}{N}\times\:dt$$.

3. MC sampling for ν:

- Count the number of pre-generated samples in the positive sample array of ν are $\:\le\:d\nu\:$.
- Update: $$\:{{\upnu\:}}_{k}\:=\:{{\upnu\:}}_{k-1}\:+\:Bound\times\:\frac{Count}{N}\times\:dt$$.

C. Variable polytropic index:

Adjust the local polytropic index *n* according to the present location in relation to the transition layer ε:

40 $$\:\:\:\:n={{\upeta\:}}_{0}-{{\upeta\:}}_{1}\bullet\:\mathrm{tanh}\left(\frac{\frac{x}{{x}_{\mathrm{s}}}-{\mathrm{x}}_{c}}{\epsilon\:}\right).$$

D. Termination criteria:

If$\:\:{x}_{\mathrm{s}}\:$approaches $\:x$ or $\:\theta\:$ falls below $\:zero$, which represents the star’s physical surface, the integration is terminated.

### B.4. Selection and statistics

- Best fit selection: Find the iteration where the final $\:\theta\:$ is closest to zero (the most precise surface boundary) among the 100 parallel iterations per run.
- Aggregation: Gather each of the *m* runs’ Best $\:\theta\:$ Point.
- Statistical Analysis: To guarantee model stability, compute the Mean and Standard Deviation for the final radius ($\:x$), mass ($\:{\upnu\:}$), density ($\:\theta\:$) and the composite polytropic index *n* for all runs.

### B.5. Data export

- Complete trajectory: Save all m runs’ integration paths ($\:x$, $\:\theta\:$, $\:{\upnu\:}$, *n*, *run*) to a CSV file.
- Summary data: To a different summary CSV file, store the final surface parameters ($\:{x}_{\mathrm{s}}$, $\:x$, $\:\theta\:$, $\:{\upnu\:}$, *n*).

### B.6. Generating mean profiles algorithm

This approach is crucial for developing an integrated “Mean Model” of the neutron star since, based on the previous algorithm, the stochastic simulations yield varied step counts and surface boundaries $\:{x}_{\mathrm{s}}$.

1. Data import and structural diagnosis

- Multi-sheet extraction: Open the main spreadsheet containing the findings of the *m* independent simulation runs.
- Variable mapping: To map simulation outputs to physical variables, adopt a standardised internal column name convention.
- $\:x$: Independent radial variable (dimensionless radius).
- $\:\theta\:$: Dependent variable for normalised density.
- $\:{\upnu\:}$: Dependent variable for dimensionless mass.
- *n*: The local polytropic index profile.

2. Grid construction.

- Boundary determination: To establish the global domain, find the absolute minimum and maximum radial values $\:\left[{x}_{min},{x}_{max}\right]$ for each of the *m* runs.
- Uniform grid generation: Create a standardised linear grid with 1,000 equally spaced points $\:{x}_{grid}$. This guarantees a direct comparison of data from runs with varying step sizes.

3. Linear interpolation and resampling

- Trajectory alignment: Map the original $\:\theta\:$, $\:{\upnu\:}$ and *n* values onto the standardised 1,000-point $\:{x}_{grid}$ for each of the *m* simulation runs using a linear interpolation function (*approx*).
- Rule Handling: Maintain a continuous data frame for each run by using constant extrapolation (rule 2) to prevent runs that terminate earlier than others from producing null values.

4. Statistical moment calculation

- Ensemble averaging: Compute the arithmetic mean µ for $\:\theta\:$, $\:{\upnu\:}$ and *n* at every point on the standardised grid:

41 $$\:\stackrel{-}{f}\left({x}_{i}\right)=\frac{1}{m}\sum\:_{run=1}^{m}{f}_{run}\left({x}_{i}\right).\:$$

- Variance analysis: Calculate the Standard Deviation σ for the density profile $\:\theta\:$ to quantify the uncertainty/stability introduced by the stochastic MC solver.

5. Result consolidation and export

- Summary compilation: Create a single “Mean Distribution” table by combining the grid and mean values.
- Multi-level reporting: Create an extensive Excel spreadsheet that includes:
- The Global Mean Distributions’ principal sheet.
- For detailed verification, separate sheets for every one of the m interpolated runs.
- Persistence: For later astrophysical visualization and analysis, save the completed data to the local file system.

## References

[1] Shapiro, S. L. & Teukolsky, S. A. *Black Holes, White Dwarfs and Neutron Stars: The Physics of Compact Objects*10.1002/9783527617661 ( Wiley, 1983).

[2] Bicak, J. Einstein equations: exact solutions. *Encyclopaedia Math. Phys.* **2**, 165–173 (2006).

[3] Haensel, P., Zdunik, J. L. & Schaefer, R. Strange quark stars. *Astrophys. J.* **160**, 121–128 (1986).

[4] Kumar, J. & Bharti, P. Relativistic models for anisotropic compact stars: A review. *NewAR* **95**, 101662. 10.1016/j.newar.2022.101662 (2022).

[5] Karttunen, H., Kröger, P., Oja, H., Poutanen, M. & Donner, K. J. Fundamental Astronomy. 1987, Springer Study Edition. Springer, New York, NY.

[6] Chandrasekhar, S. *An Introduction to the Study of Stellar Structure* (University of Chicago, 1939).

[7] Tooper, R. General Relativistic Polytropic Fluid Spheres. *Astrophys. J.* **140**, 434 (1964).

[8] Saad, A. N. S., Nouh, M. I., Shaker, A. A. & Kamel, T. M. Stability of the Relativistic Polytropes. *RMxAA* **57**, 407. 10.22201/ia.01851101p.2021.57.02.13 (2021).

[9] Abellán, G., Fuenmayor, E. & Herrera, L. The double polytrope for anisotropic matter: Newtonian Case. *Phys. Dark Univ.* **28**, 100549 (2020).

[10] Herrera, L. & Barreto, W. Newtonian polytropes for anisotropic matter: General framework and applications. *Phys. Rev. D*. **87**, 087303 (2013).

[11] Herrera, L. & Barreto, W. General relativistic polytropes for anisotropic matter: The general formalism and applications 2013. *Phys. Rev. D* **88**, 084022 .

[12] Herrera, L., Di Prisco, A., Barreto, W. & Ospino J. Conformally flat polytropes for anisotropic matter. *Gen. Relativ. Gravit.* **46**, 1827 (2014).

[13] Nouh, M. I. et al. White Dwarfs as a Polytropic Gas Sphere. *Astrophys* **59**, 540. 10.1007/s10511-016-9456-3 (2016).

[14] Astashenok, A. V., Odintsov, S. D. & Oikonomou, V. K. Maximal masses of white dwarfs for polytropes in *R*2 gravity and theoretical constraints. *Phys. Rev. D*. **106**, 124010 (2022).

[15] Aboueisha, M. S., Nouh, M. I., Abdel-Salam, E. A. B., Kamel, T. M. & Beheary, M. M. Gadallah K.A.K. Analysis of the Fractional Relativistic Polytropic Gas Spheres. *Sci. Rep.* **13**, 14304. 10.1038/s41598-023-41392-y (2023). 37652937 10.1038/s41598-023-41392-y PMC10471581

[16] Chavanis, P. H. & Harko, T. Bose-Einstein condensate general relativistic stars. *Phys. Rev. D*. **86**, 0640110 (2012).

[17] Lattimer, J. M. & Prakash, M. The equation of state of hot, dense matter and neutron stars. *Phys. Rep.* **621**, 127 (2016).

[18] Hebeler, K. et al. Equation of State and Neutron Star Properties Constrained by Nuclear Physics and Observation. *Astrophys. J.* **773**, 11 (2013).

[19] Özel, F., Freire, P. & Masses Radii, and the Equation of State of Neutron Stars. *Ann. Rev. A*. **54**, 401 (2016).

[20] Steiner, A. W. et al. Constraining the mass and radius of neutron stars in globular clusters. *Mon Not R Astron. Soc.* **476**, 421 (2018).

[21] Nouh, M. I., Foda, M. M. & Aboueisha, M. S. Compact stars with non-uniform relativistic polytrope, *Scii. Rep.*, vol. 14, Art. no. 16237, 10.1038/s41598-024-65973-7 (2024). 10.1038/s41598-024-65973-7 PMC11247101 39004673

[22] Nasheeha, R. N., Thirukkanesh, S. & C. Ragel, F. Core-envelope polytropic star with distinct polytropic indexes. *InJPh tmp*. 10.1007/s12648-023-02857-y (2023).

[23] Mathias, A. V., Sunzu, J. M. & Mkenyeleye, J. M. Double-Layered Anisotropic Stellar Model of Embedding Class I with Gaseous Envelope. *NewA* **106**, 102115 (2024).

[24] Olengeile, L., Sunzu, J. M. & Mkenyeleye, J. M. Three-layered super dense star with charged anisotropic fluid. *NewA* **110**, 102229. 10.1016/j.newast.2024.102229 (2024).

[25] Lighuda, A. S., Maharaj, S. D., Sunzu, J. M. & Mureithi, E. W. Three-layered star comprising polytropic, quark and gaseous matter. *Prama* **97**, 5. 10.1007/s12043-022-02475-z (2023).

[26] Sunzu, J. M. & Lighuda, A. S. A generalised double layered model with polytropic and quadratic equations of state. *NewA* **100**, 101977. 10.1016/j.newast.2022.101977 (2023).

[27] Liu, S. F., Guillochon, J., Lin, D. N. C. & Ramirez-Ruiz, E. On the Survivability and Metamorphism of Tidally Disrupted Giant Planets: The Role of Dense Cores. *ApJ* **762** (37). 10.1088/0004-637X/762/1/37 (2013).

[28] Criss, R. E. & Hofmeister, A. M. Analytical representations for simple and composite polytropes and their moments of inertia. *NewA* **36** (26). 10.1016/j.newast.2014.09.012 (2015).

[29] ; Steve Brooks; Andrew Gelman, Galin, L. & Jones Xiao-Li Meng; Handbook of Markov Chain Monte Carlo, (2011).

[30] Daniel Foreman-Mackey, David, W. & Hogg *Dustin Lang; Jonathan Goodman* (The MCMC Hammer, ARXIV-ASTRO-PH.IM, 2012).

[31] Thomas, A. *Ottosen; Markov-Chain Monte-Carlo A Bayesian* (Approach To Statistical Mechanics, ARXIV-ASTRO-PH.GA, 2012).

[32] ; David Parkinson, Andrew, R., Liddle & Review, A. Bayesian Model Averaging In Astrophysics: ARXIV-ASTRO-PH.IM, (2013).

[33] van Rutger, H. Marginal Likelihood Calculation with MCMC Methods, (2014).

[34] Geetakrishnasai Gunapati; Anirudh Jain & Srijith, P. K. *Shantanu Desai; Variational Inference As and Alternative to MCMC for Parameter Estimation and Model Selection* (ARXIV-ASTRO-PH.IM, 2018).

[35] Daniel, F. M. et al. *Nelson; Miguel de Val-Borro; Tobias Erhardt; Ilya Pashchenko; Oriol Abril Pla; Emcee V3: A Python Ensemble Sampling Toolkit For Affine-invariant MCMC* (ARXIV-ASTRO-PH.IM, 2019).

[36] Gregory Ashton. *Colm Talbot; Bilby-MCMC: An MCMC Sampler for Gravitational-wave Inference* (ARXIV-GR-QC, 2021).

[37] Joshua Albert; Csaba Balazs. *Martin White; A Comparison of Bayesian Sampling Algorithms for High-dimensional Particle Physics* (and Cosmology Applications, ARXIV-HEP-PH, 2024). Andrew Fowlie; Will Handley; Nicholas Hunt-Smith; Roberto Ruiz de Austri.

[38] Bo Liang; Hanlin Song. *Ziren Luo; Estimating Orbital Parameters of Direct Imaging Exoplanet Using Neural Network* (ARXIV-ASTRO-PH.EP, 2025).

[39] El-Essawy, S. H., Nouh, M. I., Soliman, A. A., Rahman, A. & Abd-Elmougod, G. A. H. I., and Monte Carlo simulation of Lane-Emden type equations arising in astrophysics, Astronomy and Computing, vol. 42, Art. no. 100665, Elsevier, 10.1016/j.ascom.2022.100665 (2023).

[40] El-Essawy, S. H., Nouh, M. I., Soliman, A. A., Rahman, A. & Abd-Elmougod, G. A. H. I., and A novel numerical solution to lane-emden type equations using monte carlo technique, Physica Scripta, vol. 99, no. 1, Art. no. 015224, IOP, 10.1088/1402-4896/ad137b (2024).

[41] Nouh, M. I., Elkholy, E. A. & El-Essawy, S. H. Computing Polytropic and Isothermal Models Using Monte Carlo Method, Revista Mexicana de Astronomia y Astrofisica, vol. 60, pp. 3–12, 10.22201/ia.01851101p.2024.60.01.01 (2024).

[42] El- Essawy, S., Soliman, A., Nouh, M., Abdel- Rahman, H. & Abd- Elmougod, G. A New Numerical Solution to the Lane- Emden Equations with Cylindrical and Planar Polytropes Using the Monte Carlo Method. *Sohag J. Sci.* **9** (4), 551–571. 10.21608/sjsci.2024.283603.1197 (2024).

[43] Wei, X. Construct a realistic stellar model with polytropic relation. *A&C* **41**, 100650. (2022).

[44] Mathias, A. V., Sunzu, J. M. & Mkenyeleye, J. M. Double-layered anisotropic stellar model of embedding class I with gaseous envelope. *NewA* **106**, 102115 (2024).

[45] Olengeile, L., Sunzu, J. M. & Mkenyeleye, J. M. Three-layered super dense star with charged anisotropic fluid. *NewA* **110**, 102229. 10.1016/j.newast.2024.102229 (2024).

[46] Chandrasekhar, S. The dynamical instability of gaseous masses approaching the schwarzschild limit in general relativity. *Astrophys. J.* **140**, 417. 10.1086/147938 (1964).

[47] Heintzmann, H. & Hillebrandt, W. Neutron stars with an anisotropic equation of state: Mass, redshift and stability. *AAP* **38**, 51 (1975).

[48] Zeldovich, Y. B. & Novikov, I. D. Relativistic Astrophysics. Stars and Relativity Vol. 1 University of Chicago Press, (1971).

[49] Abubekerov, M. K., Antokhina, E. A., Cherepashchuk, A. M. & Shimanskii, V. V. Impact of Rastall gravity on mass, radius, and sound speed of the pulsar PSR J0740+ 6620 *ARep*, **52**, 379. (2008).

[50] Gangopadhyay, T., Ray, S., Li, X. D., Dey, J. & Dey, M. Strange star equation of state fits the refined mass measurement of 12 pulsars and predicts their radii *MNRAS*, 431, 3216. (2013).

[51] Rawls, M. L. et al. *ApJ*, **730**, 25. (2011).

[52] Naik, S., Paul, B. & Ali, Z. Refined neutron star mass determinations for six eclipsing x-ray pulsar binaries*ApJ*, **737**, 79. (2011).

[53] Özel, F., Guver, T. & Psaltis, D. The mass and radius of the neutron star in EXO 1745- 248 *ApJ*, **693**, 1775. (2009).

[54] Webb, N. A. & Barret, D. Constraining the equation of state of supranuclear dense matter from XMM-newton observations of neutron stars in globular clusters *ApJ*, **671**, 727. (2007).

[55] Bogdanov, S., Heinke, C. O., Özel, F. & Güver, T. Neutron star mass--radius constraints of the quiescent low-mass X-ray binaries X7 and X5 in the globular cluster 47 Tuc *ApJ*, **831**, 184. (2016).

[56] Özel, F. et al. The dense matter equation of state from neutron star radius and mass measurements *ApJ*, **820**, 28. (2016).

[57] Marshall, F. E. & Angelini, L. *IAU Circ.*, **6331**, 1. (1996).

[58] Raaijmakers, G. et al. A NICER view of PSR J0030+ 0451: Implications for the dense matter equation of state *ApJL*, **887**, L22. (2019).

[59] Miller, M. C. et al. PSR J0030+ 0451 mass and radius from NICER data and implications for the properties of neutron star matter *ApJL*, **887**, L24. (2019).

[60] Reardon, D. J. et al. MNRAS, 455, 1751. (2016).

[61] Arzoumanian, Z. et al. Timing analysis for 20 millisecond pulsars in the Parkes Pulsar Timing Array *ApJS*, **235**, 37. (2018b).

[62] Antoniadis, J. et al. The NANOGrav 11-year data set: High-precision timing of 45 millisecond pulsars *Sci*, **340**, 6131. (2013).

[63] Romani, R. W. et al. : The Fastest and Heaviest Known Galactic Neutron Star, *ApJ* vol. 934, no. 2, Art. no. L17, IOP, 10.3847/2041-8213/ac8007 (2022).

[64] Abbott, R. et al. The LIGO Scientific Collaboration and The Virgo Collaboration *ApJL*, 896, L44. (2020b).

[65] Abbott, B. P. et al. Macfoy, s. and reid, s. and tokmakov, kv, ligo scientific collaboration, virgo collaboration (2018) gw170817: Measurements of neutron star radii and equation of state. physical review letters, 121. issn 0031-9007 * PHRvL*, 121, 161101. (2018). 10.1103/PhysRevLett.121.161101 30387654

[66] Demorest, P. et al. A two-solar-mass neutron star measured using Shapiro delay. *Nature* **467**, 1081–1083. 10.1038/nature09466 (2010). 20981094 10.1038/nature09466

[67] Fonseca, E. Refined Mass and Geometric Measurements of the High-mass PSR J0740 + 6620, *APJ* vol. 915, no. 1, Art. no. L12, IOP, 10.3847/2041-8213/ac03b8 (2021).

[68] Miller, M. C. The Radius of PSR J0740 + 6620 from NICER and XMM-Newton Data, *APJ* vol. 918, no. 2, Art. no. L28, IOP, 10.3847/2041-8213/ac089b (2021).

[69] Oppenheimer, J. R. & Volkoff, G. M. On Massive Neutron Cores, *Phys. Rev.* 55.374. (1939).

[70] Rhoades, C. E. & Ruffini, R. Maximum Mass of a Neutron Star, *Phys. Rev. Lett.*, vol. 32, no. 6, pp. 324–327, 10.1103/PhysRevLett.32.324 (1974).

[71] Kalogera, V. & Baym, G. The Maximum Mass of a Neutron Star. *Astrophys. J.* **470, IOP, p. L61**, 10.1086/310296 (1996).

[72] Typel, S., Röpke, G., Klähn, T., Blaschke, D. & Wolter, H. H. Composition and thermodynamics of nuclear matter with light clusters, *Phys. Rev.* C, vol. 81, no. 1, Art. no. 015803, 10.1103/PhysRevC.81.015803 (2010).

[73] Glendenning, N. K. & Moszkowski, S. A. Reconciliation of neutron-star masses and binding of the Lambda in hypernuclei, *Phys. Rev.*, vol. 67, pp. 2414–1417, 10.1103/PhysRevLett.67.2414 (1991). 10.1103/PhysRevLett.67.2414 10044420

[74] Douchin, F. & Haensel, P. A unified equation of state of dense matter and neutron star structure, *Astronom. Astrophys.*, vol. 380, EDP, pp. 151–167, 10.1051/0004-6361:20011402 (2001).

[75] Akmal, A., Pandharipande, V. R. & Ravenhall, D. G. Equation of state of nucleon matter and neutron star structure, *Phys. Rev.* C, vol. 58, no. 3, APS, pp. 1804–1828, 10.1103/PhysRevC.58.1804 (1998).

[76] Lalazissis, G. A., König, J. & Ring, P. New parametrization for the Lagrangian density of relativistic mean field theory, *Phys. Rev.* C, vol. 55, no. 1, pp. 540–543, 10.1103/PhysRevC.55.540 (1997).

[77] Read, J. S., Lackey, B. D., Owen, B. J. & Friedman, J. L. Constraints on a phenomenologically parametrized neutron-star equation of state. *Phys. Rev. D*. **79, no., APS**, 10.1103/PhysRevD.79.124032 (2009).

